# Role of Exercise in Modulating the Brain–Heart Axis in Cardiovascular Diseases

**DOI:** 10.3390/ijms27073292

**Published:** 2026-04-05

**Authors:** Wenyan Bo, Qingxiang Guo, Yixuan Ma

**Affiliations:** 1 Institute of Physical Education, Shanxi University, Taiyuan 030006, China; 2 Institute of Sports and Exercise Biology, Shaanxi Normal University, Xi’an 710119, China

**Keywords:** cardiovascular diseases, brain–heart axis, exercise, cardioprotection

## Abstract

Regular exercise is a cornerstone of the primary and secondary prevention of cardiovascular diseases (CVDs), yet the mechanisms underlying its cardioprotective effects remain incompletely understood. Emerging evidence indicates that dysfunction of the brain–heart axis is closely involved in the onset and progression of CVDs, whereas exercise may exert cardiovascular protection, at least in part, by modulating this bidirectional regulatory network. This review summarizes the pathophysiological significance of brain–heart axis dysregulation in CVDs and systematically discusses the potential mechanisms through which exercise regulates this axis to alleviate CVDs. We further examine how exercise prescription-related variables may differentially influence brain–heart axis regulation and address the potential risks associated with excessive exercise. Finally, we highlight future directions for translating these mechanistic insights into precision exercise medicine. Overall, this review provides an updated framework for understanding how exercise modulates the brain–heart axis in CVDs and offers new perspectives for their prevention and treatment.

## 1. Introduction

Cardiovascular diseases (CVDs) remain among the leading causes of global mortality, imposing substantial social and economic burdens [1]. Despite significant advances in diagnostics, pharmacological therapies, and surgical interventions, the prevalence and mortality of CVDs continue to rise, underscoring the urgent need for novel therapeutic strategies that target both underlying and exacerbating mechanisms. Regular physical exercise is a cornerstone of both primary and secondary prevention of CVDs [2]. Although the cardioprotective effects of exercise are well established, the precise biological mechanisms remain incompletely understood. Previous studies have demonstrated that exercise enhances cardiac function through multiple peripheral mechanisms, including suppression of inflammation, oxidative stress, and apoptosis, as well as promotion of angiogenesis [3,4]. In recent years, research has expanded beyond the heart itself to include remote organ systems, immune modulation, and central nervous system (CNS) involvement [5,6,7]. This has led to a growing research focus on the bidirectional communication between the brain and the heart, collectively termed the “brain–heart axis.”

The brain–heart axis comprises integrated neural (autonomic), neuroendocrine (e.g., hypothalamic–pituitary–adrenal axis, renin–angiotensin–aldosterone system), and immune-inflammatory pathways that enable reciprocal regulation between the CNS and the cardiovascular system [8,9]. Mounting evidence underscores its critical role in maintaining physiological homeostasis and contributing to disease pathogenesis [9,10,11]. Disruptions within this axis are associated with reciprocal dysfunction, whereby central nervous system disorders can impair cardiac regulation, while cardiovascular diseases are linked to an increased risk of neuropsychiatric and cognitive disturbances [12,13,14,15,16]. These interactions may establish a maladaptive feedback loop that exacerbates both neurological and cardiovascular outcomes.

Emerging evidence suggests that exercise serves as a key modulator of the brain–heart axis, particularly through mechanisms that influence central autonomic regulation [17,18,19]. This positions exercise as a potential upstream intervention capable of simultaneously targeting neural and cardiovascular dysfunction. Accordingly, this review aims to synthesize current evidence on how exercise alleviates cardiovascular diseases through modulation of the brain–heart axis, with the goal of advancing mechanistic understanding of exercise-induced cardioprotection and informing therapeutic development. Together, these perspectives offer a conceptual and mechanistic framework for the development of novel therapeutic strategies targeting this brain–heart axis.

## 2. Pathophysiological Mechanisms of Brain–Heart Axis in CVDs

### 2.1. Organization of the Central Cardiovascular Regulatory Network

**Figure 1:**
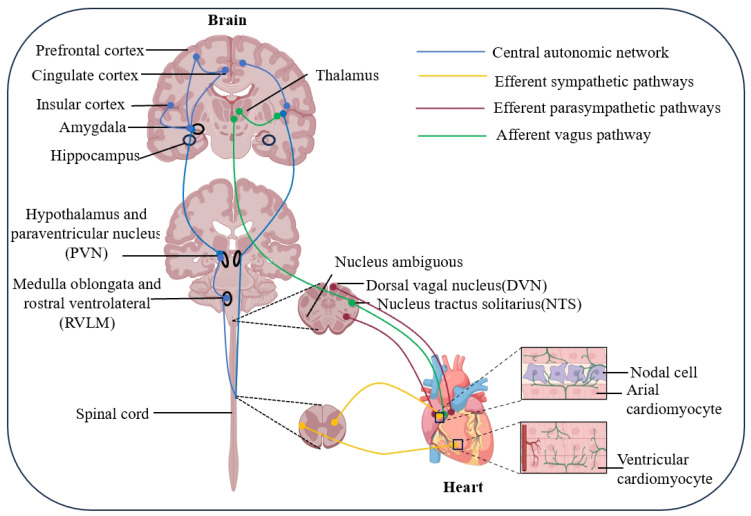
The brain–heart axis is composed of key autonomic neural networks and conduction systems. On the coronal plane of the brain, the autonomic neural network of the brain–heart axis is illustrated. Efferent pathways originate from the medial prefrontal cortex and insular cortex, terminating at cardiomyocytes and arterial endothelial cells. Afferent pathways begin with inputs from chemoreceptors and baroreceptors, ultimately projecting to higher cortical centers.

The central nervous system precisely modulates cardiac function through a multi-level, highly integrated network, which holds significant physiological and clinical implications within the framework of “brain–heart” interactions. Neuronal nuclei regulating cardiovascular activity are widely distributed from the spinal cord to the cerebral cortex, forming an intricate network encompassing the brainstem, hypothalamus, limbic system, and cortical regions. This network includes the medial prefrontal cortex (mPFC), insular cortex (IC), amygdala, bed nucleus of the stria terminalis (BNST), hypothalamic paraventricular nucleus, parabrachial area, and multiple nuclei in the medulla oblongata [12]. This hierarchical organization enables the integration of emotional, cognitive, and interoceptive signals to produce coordinated autonomic output (Figure 1). Under physiological conditions, this hierarchical organization enables flexible and coordinated regulation of sympathetic and parasympathetic outflow, whereas under pathological conditions, dysfunction at multiple levels of this network contributes to sustained autonomic imbalance and progressive cardiovascular injury.

#### 2.1.1. Cortical and Limbic Integration of Cardiovascular Signals

Although cardiovascular regulation is largely mediated by the autonomic nervous system, emerging evidence highlights the involvement of cortical areas in both physiological and pathological contexts [20,21,22]. The prefrontal cortex (PFC) has been reported to increase blood pressure through sympathetic nerve activation in response to emotional and stressful stimuli [23]. Notably, post-MI rodents exhibit elevated oxidative stress and neuroinflammatory markers in the medial prefrontal cortex (mPFC), accompanied by depressive-like behaviors [24]. Importantly, suppressing mPFC activity in post-MI rats impairs their ability to discriminate between neutral and aversive stimuli, leading to inappropriate sympathetic activation in response to benign environmental cues [25].

The insular cortex (IC), as a key component of the central autonomic network, also plays a critical role in cardiac control [26,27]. The IC exhibits functional lateralization, with the right IC being more closely associated with sympathetic cardiovascular regulation, whereas the left IC is more involved in parasympathetic modulation [28]. The IC is also implicated in stroke–heart syndrome, which encompasses various forms of stroke-induced cardiac dysfunction attributable to dysregulation of the brain–heart autonomic axis [29,30]. Direct stimulation of the IC causes a stronger cardiac response, including bradycardia and tachycardia [31]. Emerging interventions, such as transcranial direct current stimulation (tDCS) may reduce perceived exertion in HF patients by influencing IC activity, alter the quantification of inflammatory cytokines, and promote the patient’s cardiac rehabilitation process [32]. These evidence highlighting its importance in linking cerebral injury to cardiac dysfunction.

Cortical areas related to movement have also been reported to be associated with cardiovascular regulation [20,21]. Li et al. identified anatomical connections between neurons in the primary motor cortex M1 and the heart [33]. Bo et al. demonstrated that primary motor cortex M1 neurons influenced cardiac function in normal and MI mice via projections to the median raphe nucleus, and that chemogenetic activation of M1 L5 neurons worsens cardiac function post-MI [6]. Notably, recent evidence indicates that the excitability of glutamatergic neurons in the M1L5 region is abnormally elevated after MI, and suppressed premature ventricular contractions (PVCs) after MI by activating the primary motor cortex M1-ZI-RVLM neural circuit [34], further underscoring the role of M1 neurons in cardiac regulation. Emerging work also suggests that cortical regions, including M1, can exert autonomic control over cardiovascular activity during physical exertion [35]. This evidence indicates that the cerebral cortex (including the prefrontal cortex, insular cortex, motor cortex, among others) not only participates in the fine regulation of cardiovascular activity under physiological conditions but also plays a critical role in pathological processes such as myocardial ischemia.

The hippocampus, belonging to the limbic system of the brain, is closely associated with cardiovascular activity [36]. Stimulation of the hippocampus alters heart rate and blood pressure [37], whereas MI induces hippocampal neuroinflammation, oxidative stress, and neuronal injury, which are associated with cognitive impairment and depression-like behaviors after cardiac injury [38,39,40]. Likewise, the central nucleus of the amygdala (CeA), a region involved in anxiety and emotional processing, is also linked to cardiovascular regulation [41]. Experimental activation of the CeA increases arterial pressure and heart rate [42], and MI is accompanied by enhanced neuronal activity, inflammation, and apoptosis in this nucleus [43,44]. Collectively, these findings indicate that cortical and limbic regions do not merely influence emotional responses, but also provide important upstream control over cardiovascular function by integrating psychological and visceral information.

#### 2.1.2. Hypothalamic Coordination of Autonomic and Neuroendocrine Output

The hypothalamus serves as a major integrative center linking higher cortical and limbic inputs to downstream autonomic and neuroendocrine responses. Among hypothalamic nuclei, the paraventricular nucleus (PVN) is particularly important in the regulation of cardiovascular function [45,46]. The PVN receives signals from the cortex, limbic system, and peripheral circulation, and in turn projects to sympathetic preganglionic neurons and brainstem autonomic nuclei, thereby coordinating sympathetic outflow and neuroendocrine activation [47,48]. In pathological conditions such as HF and MI, PVN neuronal activity is significantly enhanced, contributing to sympathoexcitation, ventricular arrhythmias, and adverse cardiac remodeling [47,48,49].

Importantly, the PVN is not only a sympathetic integrative center but also a regulator of parasympathetic activity. Oxytocinergic (OXT) neurons in the PVN provide direct excitatory projections to parasympathetic cardiac vagal neurons (CVNs) in the brainstem. Specifically, targeted enhancement of PVN-OXT neuronal activity augments downstream CVN activity in the brainstem, thereby increasing cardiac parasympathetic drive [50]. In animal models of HF, PVN-OXT neuronal activity is reduced, while chronic chemogenetic stimulation of these neurons significantly improves left ventricular (LV) electrophysiology and contractile function, reduces hypertrophy and fibrosis, and enhances LV sensitivity to β-adrenergic stimulation [50,51,52]. Moreover, during the early phase of acute MI, chemogenetic activation of PVN-OXT neurons improves cardiac mitochondrial function and suppresses myocardial fibrosis [53]. These observations suggest that the hypothalamus, particularly the PVN, acts as a central hub coordinating both autonomic and neuroendocrine outputs during cardiovascular stress.

#### 2.1.3. Brainstem Execution of Cardiovascular Reflexes

The nucleus tractus solitarius (NTS) is a central hub for cardiovascular reflex regulation and serves as the first major integration site for visceral afferent information arising from arterial baroreceptors, peripheral chemoreceptors, and cardiopulmonary receptors. Rather than functioning as a passive relay, the NTS dynamically processes incoming signals and distributes them to downstream autonomic nuclei, including the dorsal motor nucleus of the vagus (DMV), nucleus ambiguus (Amb), and ventrolateral medulla, thereby coordinating cardiovagal reflexes, sympathetic inhibition, and short-term blood pressure stabilization [54,55]. In cardiovascular disease, however, the integrative function of the NTS becomes maladaptively remodeled. In heart failure (HF), impaired baroreflex sensitivity is associated not only with altered peripheral receptor input but also with defective central processing within the NTS. Reduced BDNF/TrkB signaling in this region contributes to diminished vagal baroreflex sensitivity and enhanced sympathetic activation [56]. In addition, pathological conditions such as chronic intermittent hypoxia can augment chemosensory afferent signaling to the NTS and trigger local neuroinflammation, characterized by transient astrocyte activation, sustained microglial activation, and increased expression of pro-inflammatory cytokines, including interleukin-6 (IL-6) and interleukin-1β (IL-1β), thereby contributing to neurogenic hypertension [57]. Experimental evidence further suggests that oxidative stress-related signaling within the NTS participates in autonomic dysregulation. For example, microinjection of chemerin-9 into the NTS increases sympathetic outflow, blood pressure, and heart rate through CMKLR1-mediated NADPH oxidase activation and superoxide production [58]. After MI, increased expression of vasopressin 1a (V1a) receptors and GABAA receptor γ2 subunits increases in the NTS further influences autonomic balance [59].

Another key brainstem region is the rostral ventrolateral medulla (RVLM), which plays a central role in maintaining resting sympathetic tone and blood pressure. Accumulating evidence indicates that oxidative stress within the RVLM contributes to sympathoexcitation in hypertension and HF [60,61,62]. Consistently, enhancement of NRF2 signaling in the RVLM alleviates oxidative stress and improves autonomic regulation, whereas Nrf2 deficiency induces hypertension and sympathetic overactivation [63,64]. In addition, catecholaminergic C1 neurons in the RVLM directly project to sympathetic preganglionic neurons and are critically involved in maladaptive autonomic responses in HF and myocardial ischemia/reperfusion injury [65,66,67,68]. Experimental inhibition or ablation of these neurons reduces cardiac sympathoexcitation, attenuates arrhythmogenesis, and improves cardiac outcomes in disease models [67,68]. Together, these findings indicate that the brainstem is not merely an execution site for autonomic output, but a critical substrate in which pathological cardiovascular reflexes are integrated and amplified.

### 2.2. Functional Remodeling of Central Autonomic Circuits in CVDs

In CVDs, the central cardiovascular regulatory network undergoes marked functional remodeling. Rather than remaining a stable controller of autonomic output, the brain becomes an active participant in disease progression. Cardiac injury sends abnormal afferent signals to the central nervous system, while circulating neuroendocrine and inflammatory factors further alter neuronal excitability and network integration. These changes reshape afferent–central–efferent autonomic circuits and establish a pathological feedback loop between the heart and the brain.

#### 2.2.1. Altered Afferent Signaling from the Injured Heart to the Brain

Myocardial ischemia, hypoxia, and necrosis lead to the release of inflammatory mediators and metabolites such as adenosine and lactate, which activate cardiac sensory receptors distributed in the epicardium and myocardium. This afferent signaling is mediated not only by classical chemosensitive mechanisms, but also by mechanosensitive pathways that respond to alterations in cardiac stretch, pressure, and wall stress. In this context, mechanically activated ion channels, particularly Piezo1 and Piezo2, have recently been recognized as important mediators of cardiac mechanotransduction, enabling afferent neurons to detect mechanical stimuli such as stretch, pressure, and shear stress [69]. Among them, Piezo2 is predominantly expressed in sensory neurons and appears to be more directly involved in the initiation of afferent mechanosensory signaling, whereas Piezo1 is more broadly distributed and primarily links mechanical stimulation to downstream intracellular signaling cascades [70]. Notably, these channels have been implicated in cardiovascular reflex regulation, including baroreceptor activity and blood pressure homeostasis [71], suggesting their potential involvement in cardiac afferent activation under pathological condition.

A particularly important pathway in this context is the cardiac sympathetic afferent reflex (CSAR), through which sympathetic afferent fibers transmit nociceptive and mechanical signals from the injured heart to the spinal cord and higher autonomic centers. CSAR is markedly enhanced in cardiovascular disease, especially in HF [72]. Increased CSAR activity has been shown to promote ventricular arrhythmias after acute myocardial infarction (AMI) [73]. During myocardial ischemia and necrosis, sympathetic afferent fibers are strongly activated and convey danger-related signals to the central nervous system, thereby indicating ongoing cardiac injury [74,75]. Together with altered baroreceptor and chemoreceptor signaling [76], these abnormal afferent inputs provide an important trigger for central autonomic remodeling. After reaching the central nervous system, these afferent signals are integrated across multiple levels of the neuraxis. Brain regions involved in autonomic, stress, and emotional regulation, particularly the hypothalamus and amygdala, contribute to the central processing of cardiac afferent information, especially during the chronic phase of cardiovascular disease [63]. Thus, altered afferent signaling from the injured heart does not merely reflect peripheral sensory activation, but represents the initiating step in a broader process of brain–heart axis maladaptation.

#### 2.2.2. Maladaptive in Sympathetic–Parasympathetic Balance

Persistent activation of central autonomic circuits ultimately leads to a maladaptive shift toward sympathetic predominance in CVDs. As a principal brainstem center governing sympathetic vasomotor tone, the RVLM integrates excitatory input from upstream nuclei, including the NTS and hypothalamic regions such as the PVN, and translates these signals into sustained sympathetic outflow [48,62]. Under pathological conditions, prolonged aberrant afferent signaling from the injured heart increases neuronal excitability within the RVLM and related autonomic nuclei, thereby establishing a state of chronic central sympathoexcitation [49]. This persistent sympathetic activation is a defining feature of cardiovascular dysregulation in CVDs. Elevated sympathetic drive promotes continuous norepinephrine release from peripheral sympathetic terminals, particularly cardiac sympathetic nerves, resulting in tachycardia, enhanced myocardial contractility, systemic vasoconstriction, and increased myocardial oxygen demand [77]. Although these responses may initially serve a compensatory role in maintaining arterial pressure and cardiac output, sustained activation becomes maladaptive. Chronic sympathetic overactivity increases afterload through peripheral vasoconstriction and, together with neurohumoral activation such as stimulation of the renin–angiotensin–aldosterone system, further elevates preload by promoting sodium and water retention. These hemodynamic and neurohumoral alterations substantially increase cardiac workload and accelerate myocardial injury.

Beyond its immediate hemodynamic effects, sustained sympathetic hyperactivity actively contributes to disease progression. Excessive central excitation within the RVLM and PVN promotes long-term neural remodeling that stabilizes the sympathoexcitatory state, thereby perpetuating autonomic imbalance [78,79,80]. This chronic sympathetic dominance not only aggravates ventricular remodeling and cardiac insufficiency, but also increases electrical instability and susceptibility to malignant arrhythmias. Mechanistically, oxidative stress within the RVLM is an important contributor to enhanced sympathetic nerve activity in hypertension and heart failure [60,61,62], while PVN overactivation has been implicated in ventricular arrhythmogenesis and peripheral sympathetic hyperactivity after MI [49]. Therefore, in CVDs, sympathetic overactivation should not be viewed merely as a secondary adaptive response, but rather as a central pathophysiological driver that links neural circuit remodeling to adverse structural, functional, and electrophysiological outcomes [80,81].

At the same time, parasympathetic regulation is markedly weakened. Cardiac parasympathetic tone is primarily mediated by vagal efferents originating from the dorsal motor nucleus of the vagus and the nucleus ambiguus, with acetylcholine release exerting important cardioprotective effects [82]. In cardiovascular disease, impaired NTS-mediated cardiovagal reflexes and reduced vagal tone diminish this endogenous protective mechanism [54,55,56]. Following MI, redox imbalance in vagal ganglia further disrupts afferent glutamatergic signaling and contributes to vagal withdrawal, thereby increasing susceptibility to ventricular tachyarrhythmias [83]. Thus, reduced parasympathetic activity is not merely a secondary consequence of cardiac injury, but an integral component of autonomic imbalance that facilitates electrical instability and disease progression.

This autonomic imbalance is particularly important in HF and post-MI remodeling. Sustained sympathetic overactivation accelerates adverse structural and electrical remodeling, whereas parasympathetic withdrawal removes an important brake on inflammation, arrhythmia susceptibility, and hemodynamic instability. Therefore, sympathetic overactivation and vagal withdrawal should be viewed as the integrated functional output of central autonomic circuit remodeling rather than as isolated downstream abnormalities.

### 2.3. Cellular and Molecular Mechanisms Underlying CVDs Dysfunction

Beyond circuit-level changes, central autonomic dysfunction in CVDs is sustained by a series of cellular and molecular alterations, including neuroinflammation, oxidative stress, neurotransmitter imbalance, and neuroendocrine activation. These processes interact with one another to stabilize pathological patterns of neuronal excitability and autonomic output.

#### 2.3.1. Neuroinflammation and Glial Activation

Neuroinflammation represents a central mechanistic driver of brain–heart axis dysregulation in CVDs. It is characterized by microglial and astrocytic activation, increased production of pro-inflammatory cytokines, and disruption of the blood–brain barrier (BBB) [9,84]. In key autonomic nuclei such as the PVN, marked microglial activation and increased BBB permeability have been observed after cardiovascular injury [85,86]. BBB disruption facilitates the entry of circulating inflammatory mediators into the brain parenchyma, thereby amplifying local neuroinflammation [87].

Once BBB integrity is compromised, infiltrating inflammatory mediators further activate glial cells within the central nervous system. Activated microglia amplify neuroinflammation and promote astrocyte activation, while astrocytes release excessive glutamate and adenosine triphosphate (ATP), forming a feed-forward loop that enhances neuronal excitability and sustains sympathetic overactivation [88]. At the molecular level, specific inflammatory pathways are critically involved in this process. In HF models, TNF-α and IL-1β expression are significantly upregulated in the PVN, promoting sympathetic excitation via extracellular signal-regulated kinase (ERK) 1/2 signaling [89]. The Toll-like receptor 4 (TLR4)/NF-κB pathway further contributes to this process, driving persistent inflammatory signaling and increasing the risk of ventricular arrhythmias following MI [90]. In addition, inflammatory signaling in the subfornical organ (SFO), particularly via AT1R, can influence neuroinflammatory responses in the PVN [91]. Similar changes have also been reported in the NTS and RVLM, suggesting that neuroinflammation is broadly distributed across central cardiovascular regulatory circuits.

Importantly, beyond BBB disruption, emerging evidence highlights impaired glymphatic clearance as a critical and potentially more sustained mechanism underlying chronic neuroinflammation and cognitive impairment HF [92,93]. The glymphatic system facilitates cerebrospinal fluid–interstitial fluid exchange and is essential for the removal of metabolic waste products, inflammatory mediators, and neurotoxic proteins from the brain [94]. In HF, dysfunction of this clearance pathway leads to the accumulation of cytokines and metabolic byproducts within the brain parenchyma, thereby maintaining a persistent pro-inflammatory microenvironment. Notably, recent experimental evidence from a heart failure model demonstrated progressive impairment of glymphatic function, characterized by a mismatch between cerebrospinal fluid influx and interstitial solute clearance, whereas overt BBB leakage was not observed at the chronic stage examined [95]. This finding suggests that, particularly in chronic HF, impaired waste clearance may play a more dominant role in sustaining neuroinflammation than BBB disruption per se. In contrast, BBB dysfunction may contribute more prominently during early or region-specific stages of disease, such as in the PVN following MI. Therefore, glymphatic dysfunction and BBB impairment should be considered complementary but temporally distinct mechanisms within the brain–heart axis. While BBB disruption facilitates the initial entry of peripheral inflammatory signals, impaired glymphatic clearance likely represents a key mechanism responsible for the persistence and amplification of neuroinflammation during chronic cardiovascular disease progression.

#### 2.3.2. Central Oxidative Stress

Central oxidative stress is now recognized as a major mechanism sustaining autonomic dysregulation in CVDs and, importantly, as an active amplifier of cardiac injury rather than a mere local biochemical abnormality. Excessive reactive oxygen species (ROS) production within key autonomic regulatory regions, particularly the RVLM, PVN, and NTS, enhances the excitability of presympathetic neurons, impairs baroreflex function, and shifts autonomic balance toward chronic sympathoexcitation. In the RVLM, increased reactive oxygen species production contributes to sympathoexcitation in hypertension and MI [60,63]. Conversely, enhancement of Nrf2 signaling in the RVLM reduces oxidative stress, improves baroreflex function, and suppresses sympathetic activation [63,64].

Studies have shown that the burst of ROS in the hypothalamus is an important factor contributing to enhanced peripheral sympathetic excitation, and blocking central ROS can significantly reduce sympathetic nerve activity [96]. Following MI, oxidative stress levels in the PVN are significantly elevated [97], leading to excessive cardiac sympathetic activation and severely impairing cardiac function [98]. Administration of the oxygen free radical scavenger Tempol or overexpression of superoxide dismutase 1 (SOD1) in the PVN significantly reduces ROS levels in this region, effectively inhibits abnormal cardiac sympathetic excitation, and improves post-MI cardiac function [99].

Beyond the brainstem and hypothalamus, oxidative injury also extends to higher-order regulatory regions after myocardial infarction. Experimental studies have shown that MI is associated with oxidative stress, neuronal injury, and functional impairment in the prefrontal cortex and hippocampus, changes that are linked to neuroinflammation, altered behavior, and disturbed central autonomic control [24,38,39]. Because these forebrain regions modulate stress responsiveness and autonomic integration, their redox injury may further bias cardiovascular regulation toward sympathetic dominance during the chronic phase after MI. In parallel, myocardial infarction can impair redox homeostasis in vagal sensory ganglia, reducing glutamatergic afferent signaling and thereby weakening reflex parasympathetic output [83], which is an important factor in triggering ventricular tachycardia.

Central redox dysregulation may also aggravate cardiac injury by disrupting neurovascular integrity. MI-induced heart failure was shown to increase BBB disruption in the PVN, a key autonomic control region, accompanied by inflammatory activation and loss of tight-junction proteins [87]. Although BBB injury is not solely caused by ROS, oxidative stress is a recognized contributor to endothelial dysfunction and barrier breakdown in autonomic nuclei, facilitating the entry of circulating inflammatory mediators into the brain and further amplifying central sympathoexcitation [86]. Through this mechanism, central redox imbalance may indirectly worsen cardiac remodeling by allowing peripheral inflammatory stress to gain greater access to sympathoregulatory circuits. Thus, redox-sensitive signaling should be considered not merely a local biochemical abnormality, but a central mechanism linking peripheral cardiovascular injury to persistent changes in neuronal excitability and autonomic output.

#### 2.3.3. Neuroendocrine Dysregulation

In addition to neural and inflammatory mechanisms, neuroendocrine pathways further amplify brain–heart crosstalk in CVDs. The hypothalamic–pituitary–adrenal (HPA) axis is a major stress-responsive system centered on the PVN. Under stress conditions, the PVN releases corticotropin-releasing hormone (CRH) and arginine vasopressin (AVP), which in turn stimulate the synthesis and secretion of adrenocorticotropic hormone (ACTH), ultimately leading to the release of glucocorticoids (GCs) (cortisol in humans or corticosterone in rodents) from the adrenal cortex. Studies indicate that serum cortisol levels are associated with the severity of stroke and the extent of insular damage, and long-term elevation of cortisol is potentially neurotoxic and linked to increased post-stroke mortality [100]. A widely accepted mechanism for brain–heart interaction is the catecholamine surge hypothesis. HPA axis activation results in significantly elevated catecholamine levels [101], which is also a critical factor in inducing malignant cardiac remodeling and lethal arrhythmias [78,79,102].

The renin–angiotensin system (RAS) constitutes another critical neuroendocrine pathway. Elevated systemic angiotensin II (Ang II) levels resulting from increased renal renin production due to reduced perfusion and cardiac output, can access the brain via circumventricular organs and influence central autonomic nuclei. Ang II enhances sympathetic activity and inhibits vagal efferent output, thereby impacting parasympathetic tone [103,104]. In HF, angiotensin II levels and AT1R expression are increased in the PVN, where they enhance sympathetic activity, and worsen water–sodium retention and adverse ventricular remodeling [105,106]. Since multiple brain regions participate in Ang II-mediated regulation of sympathetic outflow and cardiovascular reflexes, central RAS activation should be viewed as an important humoral amplifier of pathological brain–heart axis signaling [107,108,109].

In brief, the HPA axis and RAS constitute two critical stress-activated neuroendocrine pathways that, in cardiovascular pathophysiology, collectively promote sympathetic overactivation, inflammation, and adverse cardiac remodeling, thereby forming a deleterious “neuroendocrine-cardiac” feedback loop.

## 3. Brain–Heart Axis Dysfunction Aggravates CVDs

### 3.1. Interaction Between Central Stress Response and Cardiac Load

As previously described, acute or chronic psychological and physiological stress trigger dysregulation of the HPA axis and SNS by cortical and limbic regions (e.g., amygdala, prefrontal cortex), resulting in central overactivation [43,110]. During acute stress, the sympathetic nervous system is rapidly excited, leading to a substantial release of catecholamines (e.g., norepinephrine and epinephrine). Concurrently, the HPA axis is activated, increasing cortisol secretion. If stress persists, this activated state may become chronic, resulting in sustained neuroendocrine dysfunction [111].

This central overactivation directly and significantly increases cardiac load, characterized by elevated heart rate, heightened peripheral vascular resistance, and enhanced myocardial contractility. These changes collectively lead to a substantial increase in myocardial oxygen demand. Notably, in the presence of pre-existing coronary artery stenosis, this increased oxygen demand disrupts the oxygen supply-demand balance. The limited coronary flow reserve in stenotic vessels cannot adequately augment oxygen supply. Consequently, myocardial ischemia may be triggered or worsened, potentially even precipitating sudden cardiac death [111]. The resulting myocardial ischemia, accompanied by symptoms such as chest pain and dyspnea, can act as new stressors, further exacerbating emotional disturbances (e.g., anxiety, depression) and central activation, thereby establishing a “brain–heart” vicious cycle [112].

### 3.2. Autonomic Nerve Dysfunction and Deterioration of Cardiac Function

The stress-induced neuroendocrine activation described above directly contributes to autonomic imbalance, which in turn perpetuates cardiac dysfunction. Marked autonomic imbalance, characterized by diminished vagal activity, can be observed early in the course of HF [113]. This alteration not only exacerbates the cardiac metabolic burden but also serves as a key mechanism underlying malignant arrhythmias and sudden cardiac death following MI [114,115,116].

Heart rate variability (HRV), a non-invasive biomarker of autonomic function, has emerged as a potential predictor of adverse outcomes in patients with HF [114]. HRV reflects beat-to-beat variations in heart rate, influenced by both sympathetic and parasympathetic activities [117]. Reduced HRV, indicative of autonomic dysfunction, is associated with increased mortality in various cardiac conditions, including MI and chronic HF [114]. In patients with HF with reduced ejection fraction (HFrEF), HRV parameters strongly predict outcomes related to pump failure and sudden death [116]. A meta-analysis confirms that impaired HRV is a consistent and independent predictor of mortality in HF [115]. Another large-scale follow-up study reported that autonomic dysfunction, characterized by low HRV, is associated not only with a high risk of cardiovascular disease but also with an increased risk of all-cause mortality [118]. Low multiday HRV may be an indicator of autonomic dysfunction driven by more sustained sympathetic predominance, leading to a higher heart rate, hemodynamic changes, and direct arterial vasoconstriction, thereby increasing cardiac workload [119]. Consequently, in the long term, autonomic dysfunction may contribute to pathological cardiac and arterial remodeling, further elevating the risk of ischemic events and HF [120].

Arterial baroreflex sensitivity (BRS), the quantitative relationship between changes in arterial blood pressure and corresponding heart rate responses, is another significant factor contributing to cardiovascular disease [121] and a potential facilitator of ventricular arrhythmias following ventricular ischemia [122]. BRS has been shown to predict post-myocardial infarction mortality, with reduced BRS often accompanying adverse cardiovascular outcomes [123].

Collectively, autonomic imbalance in early HF is not only a critical marker of disease progression, but the resultant decrease in vagal tone coupled with sympathetic overactivation is closely linked to malignant arrhythmias, worsening cardiac function, and mortality risk. Monitoring autonomic function through indices such as HRV and BRS provides vital information for risk stratification and prognosis assessment, while also offering a theoretical foundation for interventions targeting autonomic regulation.

### 3.3. Impact of Stress and Emotional Distress

#### 3.3.1. Acute Stress and Cardiovascular Events

Acute physiological and pathological stress can trigger a sharp rise in cortisol levels; a response closely linked to the risk of ventricular arrhythmias and MI. Under acute stress, both cardiac vagal baroreflex sensitivity and HRV are reduced, each associated with an increased risk of acute cardiac events [124]. HPA axis is activated during stress, stimulating the production and release of cortisol [125]. A surge in cortisol during acute stress can potentially induce left ventricular dysfunction, blood pressure fluctuations, ventricular arrhythmias, and MI [126]. Simultaneously, acute mental stress has been shown to impair BRS [127,128], which may establish an important mechanistic link between mental stress and acute cardiac events by fostering a pro-arrhythmic environment [129]. Clinical studies indicate that mental stress induced recurrent myocardial ischemia in female patients with a history of MI [112]. Both acute and chronic stress events increased the risk of adverse cardiovascular events and mortality [130].

Mechanistically, the PFC elevates blood pressure by driving sympathetic activation in response to emotional and stressful stimuli [23]. Baroreceptor unloading leads to decreased activity in the medial prefrontal cortex (mPFC) [131], while increased mPFC activity can influence HRV levels [132]. Importantly, a higher degree of mPFC activation during psychological stress predicts major adverse cardiovascular events in patients with coronary artery disease [133]. Furthermore, the ^18^F-fluorodexoyglucose positron emission tomography/computed tomography (PET/CT) and coronary computed tomographic angiography (CCTA) evidence also confirmed the stress (especially chronic stress) stimulates the amygdala, reinforcing its role in stress-related cardiovascular pathology [134].

#### 3.3.2. Chronic Emotional Distress: Depression and Anxiety

Emotional distress, including depression and anxiety, acts both as a contributing factor to the development and progression of CVDs and as a consequence of CVDs [135]. Substantial evidence demonstrates that post-MI depression negatively impacts a range of key health behaviors critical for reducing recurrence risk, leading to physical inactivity, poor diet, sleep disturbances, continued smoking, reduced adherence to cardiovascular medications, and lower participation in cardiac rehabilitation [136,137]. Furthermore, depression is associated with altered autonomic nervous system activity, characterized by reduced HRV, elevated resting heart rate, increased catecholamine levels, and is accompanied by enhanced inflammatory activity, increased platelet reactivity, and endothelial dysfunction [138].

**Figure 2:**
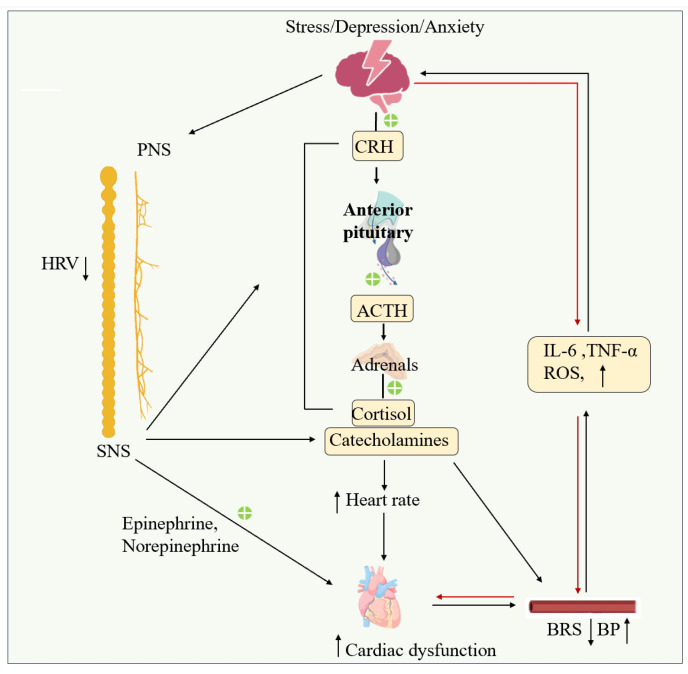
The imbalance of the brain–heart axis aggravates cardiovascular damage. Psychological stress and emotional disorders such as depression can trigger dysfunction of the HPA axis and the SNS. In this state, sympathetic overactivation leads to the excessive release of catecholamines (e.g., norepinephrine and epinephrine), accompanied by reduced HRV. Sustained activation of the HPA axis promotes the secretion of CRH and ACTH, thereby exacerbating systemic stress levels. This excessive catecholaminergic stimulation elevates heart rate and blood pressure, disrupting the myocardial oxygen supply-demand balance and consequently aggravating cardiac injury. Meanwhile, chronic HPA axis activation induces elevated expression of pro-inflammatory cytokines, which persistently damages the cardiovascular system. Furthermore, inflammatory mediators released by the injured heart, together with adverse cardiac symptoms, feedback on the central nervous system, perpetuating a detrimental heart–brain crosstalk loop. Abbreviations: ACTH adrenocorticotropic hormone, BRS baroreflex sensitivity, BP blood pressure, CRH corticotropin-releasing hormone, HRV heart rate variability, IL-6 interleukin-6, PNS parasympathetic nervous system, SNS sympathetic nervous system, ROS reactive oxygen species, TNF-α tumor necrosis factor-alpha.

Anxiety is similarly linked to various unhealthy behaviors, such as smoking, excessive alcohol consumption, physical inactivity, and poor diet, all of which elevate cardiovascular risk [139,140]. On a physiological level, acute psychosocial stress can induce coronary vasoconstriction, myocardial ischemia, and left ventricular wall motion abnormalities [130]. Studies indicate that 70% of adults with coronary artery disease experience ischemia in response to acute mental stress [141,142], which is significantly associated with an increased risk of MI, ventricular arrhythmias, and sudden cardiac death. Regarding chronic stress, prolonged sympathetic nervous system activation mediated by stress can elevate blood pressure through multiple mechanisms, including elevated C-reactive protein levels, release of inflammatory cytokines, and arterial inflammation [130]. Recognizing the importance of psychological factors, the American Heart Association and the American College of Cardiology have incorporated mental health screening and management into their latest clinical guidelines for acute coronary syndromes and cardiovascular disease prevention [143]. The bidirectional vicious cycle formed between emotional disorders and cardiovascular disease further underscores the critical importance of targeting the brain–heart axis in the prevention and management of ischemic heart disease (Figure 2).

## 4. Exercise Modulates the Brain–Heart Axis to Ameliorate Cardiovascular Disease: Focus on Central and Peripheral Mechanisms

### 4.1. Effects on Key Brain Regions and Neural Circuits

Emerging evidence from brain–heart axis research has transformed our understanding of CVDs pathophysiology, revealing that dysfunction in a distributed brain network, rather than isolated nuclei, contributes directly to conditions such as ischemic heart disease, hypertension, and HF. This network operates hierarchically: cortical and limbic regions (PFC, IC, amygdala, hippocampus) integrate emotional, cognitive, and interoceptive signals; hypothalamic nuclei (paraventricular nucleus, ventromedial hypothalamus) and circumventricular organs (subfornical organ) serve as key integrative hubs; and brainstem centers (NTS, RVM, periaqueductal gray) constitute the final common pathway for autonomic. As an important measure for the prevention and treatment of CVDs, exercise may improve cardiac function via regulating brain regions involved in cardiovascular regulation (Table 1).

The PFC serves as a critical interface between emotional processing and cardiovascular regulation. Post-MI depression and chronic stress exacerbate cardiac injury through PFC-mediated autonomic dysregulation [112,136,137]. Critically, exercise intervention mitigates these effects by reducing oxidative stress and neuroinflammation within the PFC, which correlates with amelioration of depression-like behaviors [179]. At the cellular level, MI increases the number of hypoxia-inducible factor 1-alpha (HIF-1α)-positive neurons in the rat PFC [180], suggesting a metabolic challenge that exercise may counteract. This aligns with clinical observations that HF patients exhibit reduced oxygenated hemoglobin in the PFC [181], a deficit reversed by exercise training [182]. These findings suggest that exercise preserves PFC metabolic and functional integrity under cardiovascular stress, thereby improving both emotional outcomes and autonomic balance.

The IC is a key cortical region involved in cardiovascular regulation. Under physiological conditions, the IC can inhibit sympathetic excitability in brainstem nuclei and the forebrain [183]; conversely, insular stroke disinhibits this circuitry, leading to sympathetic hyperactivity and cardiac dysfunction [183,184,185,186]. While direct evidence of exercise effects on the insula in CVDs contexts remains limited, the IC’s role in emotional processing and arrhythmogenesis positions it as a plausible target for exercise-mediated cardiovascular protection.

The primary motor cortex (M1) has recently emerged as a direct modulator of cardiac function through identified neural pathways. Glutamatergic neurons in mouse M1 regulate cardiac activity via the median raphe nucleus (MnR) [6]. More specifically, a subset of layer 5 neurons projecting directly to the RVLM mediate premature ventricular contractions following MI [34]. This M1-RVLM pathway provides a novel anatomical substrate for exercise-induced cardiovascular protection. Electroencephalography studies demonstrate that moderate exercise activates M1, whereas excessive exercise impairs motor coordination despite increasing cardiac output [187], suggesting an optimal “dose” for M1-mediated cardiac benefits. Importantly, in humans, this relationship is still at the research stage.

The hippocampus and the central nucleus of the amygdala (CeA) collectively illustrate the convergence of mood disorders and cardiovascular pathology. Stimulation of the hippocampus or CeA alter cardiovascular activity [36,188,189], and post-MI cognitive impairment predicts poor outcomes. Cognitive impairment and depression after MI are important contributors to poor outcomes, while exercise significantly improves depression-like behaviors by modulating hippocampal neuronal activity [190]. Interestingly, the effects of exercise on the hippocampus are closely related to the type of exercise and its interaction with specific brain regions [191]. Concurrently, excessive amygdala activation accelerates atherosclerosis progression through autonomic control of plaque formation [192]. Notably, the CeA is selectively activated during high-intensity exercise and promotes sympathetic drive. Therefore, for individuals at risk of or already suffering from cardiovascular disease, exercise intervention may help reduce the risk of cardiovascular events by modulating activity in the amygdala and hippocampus.

The hypothalamus plays a central role in regulating the cardiovascular system, with the PVN being a key cardiovascular center. The PVN directly modulates autonomic balance through its descending projections. Under pathological conditions, such as in aged spontaneously hypertensive rats, exercise intervention increased the density of dopamine β-hydroxylase-positive neurons and OXT neurons within the PVN, which contributed to lowering blood pressure [164]. Furthermore, exercise also improved overall cardiovascular function by reducing inflammation and oxidative stress in the PVN, which decreased central sympathetic outflow [17,18,19]. After MI, the exercise-induced reduction in Akt activity within the PVN was also considered to help ameliorate sympathetic overactivation [163].

The ventromedial hypothalamus (VMH), while classically associated with energy metabolism and emotion, significantly impacts cardiovascular regulation. VMH neuronal activation reduces heart rate and blood pressure by inhibiting medullary vasomotor neurons [166,167]. Notably, muscarinic cholinergic receptors within the VMH participate in exercise-induced cardiovascular modulation [193]. Conversely, emotional stress triggered by VMH activation increases sympathetic outflow, elevating blood pressure and exacerbating myocardial ischemia/reperfusion injury [168]. Exercise may therefore confer cardiovascular protection by stabilizing VMH responses to emotional stressors.

The subfornical organ (SFO), a highly vascularized circumventricular organ, lacks a functional blood–brain barrier. Consequently, the SFO serves as a potential interface between peripheral and central cardiovascular regulation and represents a site, where circulating peripheral molecules, such as ANG II, can access the brain. Literature has demonstrated that the SFO is neuroanatomically connected to the PVN [194]. Following HF, sympathetic nervous activity in the rat SFO increases [159,195,196]. Activation of TNF-α receptor 1 in HF rats elevates inflammatory and RAS activity in both the SFO and downstream PVN, promoting sympathetic excitation [196]. These findings suggest that targeting cytokine-receptor interactions in the SFO represents an effective “brain–heart” strategy for CVDs characterized by peripheral inflammation that a mechanism potentially engaged by exercise.

The periaqueductal gray (PAG) is a key nucleus regulating defensive behaviors and aversive emotions, and is involved in physiological processes such as cardiovascular activity, analgesia, stress responses, and memory storage. Under normal conditions, the PAG receives instructions from higher brain centers. However, under stressful conditions, it can become an initiatory center, modulating cardiovascular activity [197]. Stimulation of the PAG directly or indirectly altered myocardial contractility and modulated peripheral vascular resistance, thereby exerting dual roles in central and peripheral cardiovascular regulation [198]. Activation of the dorsolateral PAG (dl-PAG) increased sympathetic nerve activity and arterial blood pressure, and raised the firing frequency of neurons in the dl-PAG of rats with HF [174]. Twelve weeks after MI, activated microglia and neurons significantly increase in the PVN, NTS, RVLM, and PAG [199]. This distributed brainstem activation pattern suggests that exercise may improve cardiovascular function by normalizing excitability across multiple interconnected brainstem nuclei.

The RVLM and the NTS are crucial for generating and maintaining sympathetic activity, and they are key components of the central baroreflex pathway. Dopamine D1 receptors (D1R) in NTS neurons were involved in the development of stress-induced hypertension, and exercise intervention suppressed D1R expression and inhibited stress-induced hypertension [176]. Exercise training promoted beneficial neuroplasticity in the RVLM and was highly effective in restoring parasympathetic tone, attenuating age-related autonomic imbalance, and improving baroreflex function, thereby conferring long-term cardiovascular benefits for hypertensive elderly individuals [164]. Regular exercise also improves hypertension and alters gene expression in both RVLM and NTS of hypertensive rats [200]. Critically, exercise training attenuates sympathetic overactivation in mice with HF by upregulating Nrf2 expression in the RVLM [201]. These findings establish RVLM and NTS as key targets for exercise-mediated cardiovascular protection through modulation of molecular expression and neuroplasticity.

### 4.2. Enhanced Central Autonomic Regulation

Heart–brain communication via vagal pathways has been extensively demonstrated [202]. Significant autonomic imbalance, characterized by reduced vagal nerve activity, emerges early in the course of HF [113]. This imbalance not only exacerbates the cardiac metabolic burden but is also an important mechanism for malignant arrhythmias and sudden cardiac death after MI. As a non-pharmacological intervention, exercise effectively enhance cardiac vagal regulation. Clinical studies have shown that regular physical activity significantly increases HRV parameters in post-MI patients, indicating a shift in autonomic balance toward vagal predominance [203]. Exercise training also preserves vagal preganglionic neurons and restores parasympathetic tonus in HF [204]. These findings suggest that exercise partially exerts its cardioprotective effects through modulation of autonomic balance.

Following MI, catecholamine production and release are enhanced from both adrenal glands and cardiac sympathetic nerve terminals, accompanied by altered expression and function of β-adrenergic receptors [205,206]. This represents a major underlying mechanism for malignant cardiac remodeling and lethal arrhythmias [78,79,102]. Aerobic exercise significantly suppresses excessive catecholamine synthesis by downregulating G protein-coupled receptor kinase 2 (GRK2) activity in the adrenal glands [207]. An eight-week aerobic exercise regimen inhibits cardiac sympathetic nerve sprouting, restores the β3-/β1-adrenergic receptor balance, and upregulates β3-adrenergic receptor expression, thereby improving post-MI cardiac function [208]. Both aerobic and resistance training reduce cardiac sympathetic tone and improve cardiac function in post-MI mice [209]. Clinical studies further confirm that regular exercise reduces sympathetic outflow in HF patients, improving survival rates and reducing adverse cardiac events [210,211,212]. Exercise intervention also normalizes baroreflex sensitivity and muscle sympathetic nerve activity in post-MI patients, ameliorating autonomic dysfunction [213]. Taken together, these findings suggest that exercise exerts cardioprotective effects after MI partly through rebalancing autonomic output along the brain–heart axis, characterized by enhanced vagal modulation and restrained sympathetic activation, ultimately contributing to improved cardiac remodeling and prognosis.

### 4.3. Peripheral Organ Interactions That Support Exercise-Mediated Brain–Heart Axis Regulation

In addition to its direct effects on the central nervous system and the autonomic nervous system, exercise can also indirectly modulate the brain–heart axis through peripheral organs, thereby exerting cardioprotective effects. Emerging evidence indicates that the gut, skeletal muscle, spleen, and adipose tissue are not only major targets of exercise adaptation, but also key peripheral nodes involved in brain–heart communication. Exercise can remodel the gut microbiota and its metabolites, promote the release of skeletal muscle-derived exerkines and extracellular vesicles, influence spleen-mediated immune and inflammatory responses, and improve the endocrine function of adipose tissue. Through these mechanisms, exercise coordinately acts on the brain–heart axis by attenuating inflammation, maintaining metabolic homeostasis, modulating neuroplasticity, and improving autonomic balance. Therefore, peripheral organs should not be viewed merely as passive responders to exercise, but rather as active contributors to exercise-induced remodeling of the brain–heart axis and the associated cardiovascular benefits.

#### 4.3.1. The Gut–Brain–Heart Axis

The gut microbiota participates in the onset and progression of cardiac diseases via the “gut–brain” axis [214,215]. Exercise significantly affects the composition of gut microbiota and the production of metabolites, thereby regulating brain function.

MI patients often present with reduced abundance of beneficial bacteria, such as those from the Firmicutes phylum, and an increased proportion of pro-inflammatory bacteria, such as bacteroidetes and verrucomicrobia [216,217]. This imbalance leads to the accumulation of pro-inflammatory metabolites, further aggravating myocardial injury [216,218,219]. Regular physical exercise has been demonstrated to reshape gut microbiota composition, promoting the growth of beneficial species [220,221]. The intestinal barrier plays a crucial role in maintaining systemic health by preventing harmful substances from entering the circulation [214]. During HF, intestinal wall edema, reduced intestinal blood flow, decreased jejunal villus height, reduced epithelial and colonic mucosal thickness, and altered tight-junction protein expression contribute to increased intestinal permeability [222]. Exercise strengthens the intestinal barrier by improving tight-junction integrity, lowering permeability, and preventing harmful molecules from entering the bloodstream [223]. Notably, exercise reduces the Firmicutes/Bacteroidetes ratio, a marker of dysbiosis, increases acetate-producing bacterial populations, decrease lactate-producing species, and suppress the expression of pro-inflammatory genes such as TNF-α and IL-6 in the ileum. These changes improve ileal permeability, attenuate PVN microglial activation and neuroinflammation, and ultimately lower blood pressure and pressure overload–induced cardiac injury [224].

Gut microbial metabolites are key mediators linking the gut microbiota to host physiology, such as trimethylamine N-oxide (TMAO) and short-chain fatty acids (SCFAs). TMAO has been reported to stimulate sympathetic nuclei within the PVN, upregulate excitatory N-methyl-D-aspartate receptor (NMDAR) expression, activate neurons in the left stellate ganglion (LSG), and promote the expression of pro-inflammatory markers (IL-1β, IL-6, TNF-α) in this region. These effects trigger inflammatory responses, enhance cardiac sympathetic activity, and exacerbate post-ischemic ventricular arrhythmias [225]. These findings demonstrate that TMAO acts centrally to drive peripheral sympathetic outflow, exemplifying the gut–brain–sympathetic axis in cardiovascular regulation. Microbial metabolites may also modulate cardiovascular function via parasympathetic pathways. Elevated colonic acetate levels lower blood pressure through parasympathetic activation [226]. Short-chain fatty acids (SCFAs) stimulate the release of neurotransmitters and hormones, which bind to their receptors on the peripheral nervous system nerves, such as the vagus and spinal nerves, conveying information to the brain and thereby controlling blood pressure [227]. Combined exercise and low-calorie diets can significantly reduce plasma TMAO concentrations in obese adult women, thereby preventing TMAO-induced LSG activation, lowering cardiac load, and reducing arrhythmia incidence [228]. High-intensity interval training (HIIT) increases systemic acetate levels, suggesting a potential mechanism underlying the cardiovascular protective effects of HIIT [229]. Thus, exercise protects cardiac function through multiple mechanisms involving the gut–brain–heart axis, including modulation of microbial metabolites, enhancement of intestinal barrier integrity, and attenuation of neuroinflammation.

#### 4.3.2. The Muscle–Brain–Heart Axis

Skeletal muscle functions as an important endocrine organ and participates in brain–heart communication through the secretion of various myokines. Exercise-induced myokines, including irisin, insulin-like growth factor-1 (IGF-1) [230], cathepsin B (CTSB), and BDNF [231,232], act on the central nervous system to modulate autonomic function, neuroinflammation, and neuroplasticity, thereby conferring cardioprotection [233]. Importantly, the type, intensity, and duration of exercise significantly influence the expression and secretion of these myokines. For example, endurance training generally promotes the release of irisin and BDNF, whereas resistance training more prominently induces factors such as IL-6 and IL-15 [234]. Recent studies have further shown that exercise can stimulate the secretion of skeletal muscle-derived extracellular vesicles (SKM-EVs). Notably, exercise-induced SKM-EVs have been reported to promote the polarization of disease-associated microglia and enhance the clearance of amyloid-β plaques. Among their cargos, miR-378a-3p has been identified as a key microRNA enriched in SKM-EVs, which regulates lipid metabolism in disease-associated microglia by targeting p110α [235]. These findings suggest that exercise-induced SKM-EVs may act as a novel class of myokines mediating skeletal muscle–brain communication, thereby providing a potential exercise-mimetic therapeutic strategy for CVDs.

#### 4.3.3. The Spleen–Brain–Heart Axis

The spleen serves as a pivotal hub for peripheral anti-inflammatory activity and mediates brain–heart communication through immune cell trafficking, neural signaling, and cytokine release [236]. It also acts as a major reservoir for the production and storage of monocytes/macrophages [237]. After MI, a large number of monocytes and macrophages are released from the spleen and recruited to the heart via the bloodstream, directly exacerbating myocardial damage. This indicates that limiting the cardiac infiltration of splenic monocytes may represent a novel therapeutic strategy for ischemic heart injury. In models of myocardial ischemia–reperfusion injury, electrical stimulation of the splenic nerve (SpNS) significantly reduced infarct size, inflammatory response, oxidative stress, and cardiac sympathetic remodeling [238]. Mechanistically, SpNS activates the cholinergic anti-inflammatory pathway, leading to acetylcholine release in the spleen, which binds to α7 nicotinic acetylcholine receptors on splenic macrophages, suppressing pro-inflammatory cytokine production and reducing CCR2-mediated monocyte mobilization. Notably, the anti-inflammatory effects of vagus nerve stimulation depend on the integrity of the splenic nerve, as splenic nerve ablation completely blocks this effect [239], highlighting the central role of the splenic nerve in neuro-immune coupling.

Exercise intervention modulates inflammatory responses could through spleen-dependent mechanisms. Swimming exercise significantly lowered serum TNF levels in mice, an effect that was abolished in splenectomized mice [240]. Exercise also reduces circulating and splenic TNF-α levels in obese mice, alleviating insulin resistance; however, this benefit was blocked by subdiaphragmatic vagotomy [241]. These findings demonstrate that exercise-induced anti-inflammatory effects are partially mediated by the vagus nerve–splenic nerve axis. Overall, the spleen acts as a critical node in neuro-immune interactions and plays a central role in cardiac complications induced by myocardial and brain injury. Exercise may confer protection in brain–heart interactive disorders by modulating splenic-related neuro-immune pathways and attenuating systemic inflammation. Targeting the splenic nerve and its associated immunomodulatory mechanisms holds promise for developing novel therapies for cardiovascular and neuro-inflammatory diseases.

#### 4.3.4. The Brain–BAT–Heart Axis

Brown adipose tissue (BAT) is not only a core organ for thermogenesis but also a critical source of adipokines, collectively referred to as batokines. BAT expends energy in the form of heat through uncoupling protein 1 (UCP1)-mediated thermogenesis, while simultaneously coordinating its activity with systemic metabolic demands via the secretion of signaling molecules, thereby maintaining systemic energy homeostasis [242]. In recent years, the role of BAT in cardiovascular protection has garnered increasing attention.

BAT transplantation studies have demonstrated that exogenous supplementation with healthy BAT significantly ameliorates metabolic disturbances and cardiac remodeling following MI. BAT transplantation into the subcutaneous region remotely improves cardiac function, as evidenced by improved glucose tolerance, attenuated left ventricular mass increase, and enhanced exercise tolerance [243]. These findings suggest that BAT-derived circulating factors mediate these cardioprotective effects. Further studies in models of chronic ischemic cardiomyopathy have revealed a close correlation between endogenous BAT activation and left ventricular dysfunction. UCP1 deficiency, resulting in BAT dysfunction, exacerbates cardiomyocyte injury, promotes adverse left ventricular remodeling, and reduces survival rates in a mouse model of catecholamine-induced cardiomyopathy [244]. These observations underscore the importance of functional BAT in maintaining cardiac health. BAT may serve as a crucial mediator linking exercise stimulation to cardioprotective effects. Long-term exercise training (4 weeks) activates BAT, promoting the release of extracellular vesicles (EVs) carrying cardioprotective microRNAs (miRNAs), including miR-30c and miR-21, which subsequently mitigate myocardial IR injury and improve cardiac function [245]. Recent findings have further elucidated the existence and regulatory mechanisms of a “brain–adipose–heart” axis. Central leptin signaling activates hypothalamic leptin receptors (LepR), increasing sympathetic nervous system output to BAT. This stimulates BAT to secrete EVs rich in miR-29c-3p, which are taken up by cardiomyocytes. Once internalized, these EVs effectively improve mitochondrial function and inhibit cardiac fibrosis, ultimately conferring protection against ischemic heart injury [246]. This discovery underscores the role of BAT as a key functional mediator of the brain–heart axis, transducing central nervous system signals into peripheral cardioprotective effects.

**Figure 3:**
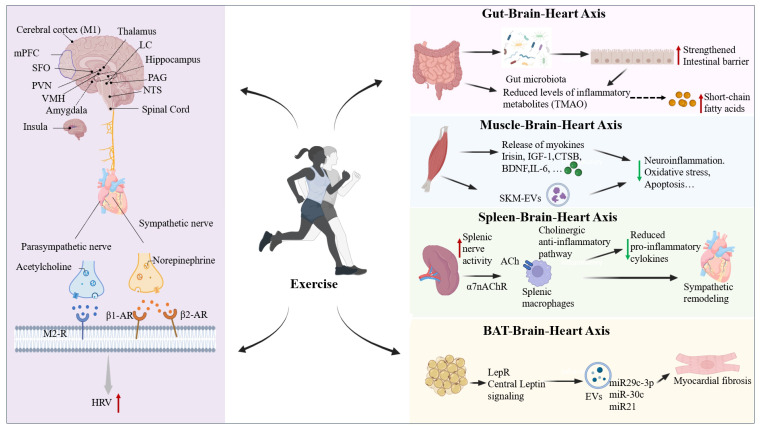
The central and peripheral mechanisms by which exercise regulates the brain–heart axis. Exercise influences key components of the central autonomic network to rebalance sympathetic and parasympathetic output, thereby regulating cardiac receptor signaling and improving heart rate variability. In parallel, exercise modulates the gut–brain–heart, muscle–brain–heart, spleen–brain–heart, and BAT–brain–heart axis, collectively reducing neuroinflammation, oxidative stress, apoptosis, myocardial fibrosis, and sympathetic remodeling, and ultimately contributing to cardiovascular protection. Abbreviations: Ach acetylcholine, α7nAChR, alpha 7 nicotinic acetylcholine receptor; β1-AR, β1-adrenergic receptor; β2-AR, β2-adrenergic receptor, BAT brown adipose tissue, BDNF brain-derived neurotrophic factor, CTSB cathepsin B, EVs extracellular vesicles, HRV heart rate variability, IGF-1 insulin-like growth factor-1, IL-6 interleukin-6, LC locus coeruleus, LepR leptin receptor, M1 primary motor cortex, mPFC medial prefrontal cortex, M2-R M2 muscarinic receptor, miR-21 microRNA-21, miR-29c-3p microRNA-29c-3p, miR-30c microRNA-30c, NST nucleus of the solitary tract, PAG periaqueductal gray, PVN paraventricular nucleus, SFO subfornical organ, SKM-EVs skeletal muscle-derived extracellular vesicles, TMAO trimethylamine N-oxide, VMH ventromedial hypothalamus.

Taken together, exercise confers cardiovascular protection through integrated modulation of the brain–heart axis. It influences key brain regions and neural circuits responsible for cardiovascular regulation, improves autonomic homeostasis by reducing sympathetic overactivation and enhancing parasympathetic tone, and further shapes brain–heart communication through the signals derived from peripheral organs such as the gut, skeletal muscle, liver, spleen, and adipose tissue. Through this coordinated central-peripheral regulatory network, exercise mitigates the development and progression of cardiovascular diseases, highlighting the brain–heart axis as a promising mechanistic framework and therapeutic target for exercise-based interventions (Figure 3).

## 5. Mechanisms Underlying the Protective Effects of Exercise Against CVDs

### 5.1. Exercise-Induced Attenuation of Neuroinflammation and Neuroimmune Responses

Exercise training significantly reduces inflammatory factor expression in both the ischemic heart and systemic circulation, thereby delaying cardiac functional deterioration [4]. This anti-inflammatory effect may be partially mediated by the vagus nerve-dependent CAIP. Numerous animal studies have demonstrated that vagus nerve stimulation effectively reduces myocardial infarct size, improves survival rates, and enhances cardiac function after MI [247,248,249,250]. These protective effects are dependent on an intact CAIP pathway [251,252]. Clinical studies have further confirmed that transcutaneous vagus nerve stimulation significantly improved inflammatory markers, cardiac function, and exercise tolerance in patients with HF with preserved ejection fraction [253]. Mechanistically, exercise enhances vagal tone [254], which subsequently inhibits the production of pro-inflammatory cytokines, such as tumor necrosis factor (TNF), in peripheral immune organs including the spleen [255]. Vagotomy abolishes the regulatory effect of exercise on serum TNF levels, indicating that the anti-inflammatory effect of exercise is partially vagus nerve-dependent [240]. The α7 nicotinic acetylcholine receptor (α7nAChR) plays a pivotal role in this pathway. Exercise alleviates lipopolysaccharide (LPS)-induced downregulation of α7nAChR expression and ameliorates neuroinflammation [256]. Conversely, administration of an α7nAChR agonist in a MI model significantly reduces inflammation [257].

Beyond peripheral immune modulation, exercise directly attenuates neuroinflammation in brain regions critical for cardiovascular control. In the PVN, aerobic exercise downregulates IL-1β and TNF-α expression, delaying blood pressure elevation in the 2-kidney, 1-clip (2K1C) renovascular hypertensive model [17]. Exercise also reduces microglial activation and pro-inflammatory cytokine expression in the PVN of spontaneously hypertensive rats (SHRs), ameliorating autonomic imbalance [18]. Furthermore, aerobic exercise decreases TNF-α and IL-6 protein levels in the PVN of hypertensive rats, inhibits microglial activation, and reverses hypertension-induced cardiac autonomic dysfunction [18].

Oxidative stress in key autonomic nuclei drives neuroinflammation and sympathetic overactivation. After MI, oxidative stress levels in the PVN are significantly elevated, leading to excessive cardiac sympathetic nerve activation and severe cardiac dysfunction [85,97]. Moderate exercise alleviates central oxidative stress in various cardiovascular conditions, including hypertension [19,258,259] and chronic HF [201]. In HF models, exercise reduces the expression of NADPH oxidase and oxidative stress in the RVLM, thereby improving autonomic imbalance and cardiac function [260]. Long-term exercise also alleviates oxidative stress and inflammation in the PVN and RVLM of spontaneously hypertensive rats, suppresses neuronal hyperexcitability, and lowers blood pressure [261]. Aerobic exercise modify synaptic transmission and ROS production in the PVN of SHRs, ameliorating abnormalities in HR and blood pressure [19], possibly by inhibiting the ROS/MAPK/NF-κB/AT1R pathway in the PVN [262]. Recent research reports that aerobic exercise alleviates neuronal oxidative stress and ferroptosis in the PVN caused by ischemia/reperfusion [165]. Collectively, these findings suggest that attenuation of oxidative stress in key autonomic nuclei represents an important mechanism by which exercise restrains neuroinflammation and sympathetic excitation, thereby protecting against cardiovascular dysfunction.

### 5.2. Exercise-Induced Promotion of Neurotrophic Support and Neuroplasticity

Neuroplasticity refers to the brain’s ability to repair connections or rewire itself structurally and functionally in response to changes in internal and external environments, including the effects of injury, drugs, environmental stress, and experience on the nervous system [164]. Accumulating evidence indicates that exercise induces structural and functional plasticity in the adult brain, encompassing angiogenesis, neurogenesis, and plasticity of dendritic morphology and synapses.

In cardiovascular disease states, maladaptive neuroplastic changes occur in key autonomic nuclei. Following MI, excitatory sympathetic input to PVN-RVLM neurons increases, and optogenetic inhibition of this pathway significantly reduces peripheral RSNA [48]. In rats with HF, the expression of synaptic plasticity-related genes is significantly reduced in brain regions critical for autonomic control, including the PVN, PFC, and CeA [263]. After exercise intervention, the number of BrdU-positive neurons, a marker of cell proliferation, increases in the PVN and hippocampus of HF rats [264]. Furthermore, exercise intervention also restores GABAA receptor function within the PVN, thereby reducing sympathetic outflow in spontaneously hypertensive rats [265]. Exercise-induced neuroplastic changes have been observed in multiple brain regions involved in cardiovascular regulation, including the NTS, posterior hypothalamus, and cortical areas [266,267]. Collectively, these findings demonstrate that exercise promotes plasticity in sympathetic-related neurons, contributing to improved autonomic balance and cardiovascular function, although the precise underlying mechanisms warrant further investigation.

Physical exercise promotes neuroplasticity, particularly in autonomic regulatory centers, through the upregulation of neurotrophic factors such as brain-derived neurotrophic factor (BDNF) [268,269,270]. BDNF is a pleiotropic neurotrophic factor that plays an important protective role in both the cardiovascular and nervous systems. Recent studies show that decreased circulating BDNF levels are significantly associated with adverse clinical outcomes in patients with HF [271], and low serum BDNF levels serve as a biomarker of poor prognosis in cardiovascular disease [272]. Peripheral BDNF concentration changes are considered partially derived from central nervous system release [273,274]. Both aerobic and anaerobic exercise influence BDNF release [275,276]. Exercise-induced neuroplastic changes in the PFC and hippocampus enhance cognitive capacity and improve autonomic regulation of cardiovascular parameters such as BP and HR [277,278]. Importantly, exercise elevates plasma BDNF levels while attenuating post-infarction activation in the PVN and RVLM, thereby suppressing cardiac sympathetic overactivation [163]. It is predicted that BDNF plays a pivotal mediating role in exercise-induced cardiac protection through peripheral cardiac signaling pathways and central sympathetic regulation. Mechanistically, BDNF binds to tropomyosin receptor kinase B (TrkB), activating downstream signaling pathways such as PI3K/Akt and MAPK/ERK, which promote neuronal survival, synaptic plasticity, and inhibit apoptosis [279,280]. These findings suggest that BDNF mediates exercise-induced cardioprotection through both peripheral cardiac signaling pathways and central sympathetic regulation.

### 5.3. Exercise-Induced Regulation of Neuroendocrine Axis

The HPA axis and the RAS constitute two critical neuroendocrine pathways whose dysregulation contributes to malignant cardiac remodeling and lethal arrhythmias following myocardial ischemic injury [78,79,102]. Exercise training modulates both axes, thereby exerting cardioprotective effects. Exercise significantly reduces adrenal catecholamine production, attenuating sympathetic overactivation [207]. In mice, cardiomyocyte-specific knockout of the glucocorticoid receptor (GR) promotes cardiac hypertrophy and progression to left ventricular systolic dysfunction [281]. Exercise training may suppress HPA axis drive by transiently altering GR gene transcription in the PVN and pituitary, as well as by modulating CRH signaling [282].

Chronic HPA axis overactivation affects gastrointestinal function and alters gut microbiota composition. Conversely, gut microbiota changes can modulate HPA axis activity, and prolonged stress-induced HPA activation may contribute to mental health disorders [283,284]. Notably, depression is an independent risk factor for coronary heart disease. Post-MI depression is also associated with increased susceptibility to all-cause mortality and cardiovascular events [285]. Physical exercise modulates the gut microbiota and increases the abundance of beneficial microbial species [220,221], suggesting that exercise may exert cardioprotective effects via the “microbiota-gut-HPA axis”. Future research is needed to further elucidate the specific mechanisms by which different exercise patterns affect the stress response of the HPA axis, and to provide evidence-based evidence for clinical applications.

Moreover, research have shown that chronic exercise modulates RAS components and improves balance between pro-and anti-inflammatory cytokines in the brain of spontaneously hypertensive rats [261]. For example, exercise training attenuates hypertension via suppressing ROS/MAPK/NF-κB/AT1 pathway in the hypothalamic paraventricular nucleus [262]. In HF rats, exercise training significantly reduces AT1 expression levels in both the PVN and the RVLM [286]. Microinjection of ANG II into the SFO of HF rats causes notable increases in blood pressure, RSNA, and HR, while exercise intervention can reverse these changes, likely through inhibition of SFO neuron activation and suppression of Ang II-mediated sympathetic excitation [159]. These findings suggest that exercise may reduce sympathetic nervous activity by downregulating AT1 expression and attenuating angiotensinergic drive. On the other hand, activation of the central RAS after HF is closely associated with an imbalance between angiotensin-converting enzyme (ACE) and ACE2. Exercise intervention can restore this balance and ameliorate ANG II-mediated excessive sympathetic excitation [287]. Acute endurance training significantly increases the plasma ACE2 level in male athletes [288], suggesting that exercise may also modulate peripheral RAS components, although the contribution of this effect to central cardioprotection requires further investigation.

### 5.4. Exerkines

#### 5.4.1. Myokines

Exercise promotes skeletal muscle secretion of myokines, thereby strengthening peripheral–central communication and contributing to neuroprotection. Among these, irisin, a cleavage product of FNDC5, is markedly upregulated during exercise and released into the circulation, where it can cross the BBB and act directly on the central nervous system [289,290]. Growing evidence indicates that exercise-induced irisin is an important mediator of the beneficial effects of exercise on the brain. It enhances synaptic stability and plasticity through CREB-dependent transcriptional programs, improves neuroimmune homeostasis by promoting microglial polarization toward the anti-inflammatory M2 phenotype via αVβ5 integrin/STAT6 signaling, and suppresses neuroinflammatory responses through inhibition of NF-κB and MAPK pathways, thereby attenuating neuronal damage [291,292,293,294]. These findings support that exercise, at least in part through the FNDC5/irisin axis, exerts coordinated effects on synaptic plasticity, inflammation, and mitochondrial homeostasis within the muscle–brain–heart axis.

IGF-1 is a polypeptide hormone primarily secreted by the liver. However, under exercise stimulation, skeletal muscle can locally synthesize and secrete IGF-1, thereby significantly elevating circulating IGF-1 levels [295,296]. This exercise-induced release of IGF-1 constitutes an important molecular basis for “muscle–brain” crosstalk. In the central nervous system, IGF-1 binds to its receptor IGF-1R and activates downstream signaling pathways that regulate neuronal growth, differentiation, survival, and functional maintenance [297,298]. IGF-1 exerts neuroprotective effects by regulating neuronal survival and synaptic plasticity. Long-term elevation of IGF-1 in animal models significantly increases dendritic complexity and spine density, enhancing information encoding efficiency and promoting long-term memory maintenance [299]. IGF-1 also inhibits central inflammation and oxidative stress. In TBI models, early upregulation of IGF-1 expression through gene therapy significantly reduces lipid peroxidation and protein oxidation in the prefrontal cortex and hippocampus, while suppressing microglia-mediated neuroinflammatory responses and ultimately improving working memory deficits [300]. In APP/PS1 mice subjected to moderate-intensity exercise, nuclear translocation of IGF-1R and SUMO1 in the hippocampus is reduced, accompanied by decreased levels of neuroinflammatory markers IL-6 and IL-1β [230]. These findings suggest that IGF-1 mediates exercise-induced muscle–brain dialogue and contributes to cardioprotection.

CTSB is a lysosomal cysteine protease that plays a crucial role in intracellular protein catabolism [301]. Exercise-induced skeletal muscle contraction stimulates the release of CTSB into the bloodstream. Studies demonstrate that CTSB levels increase following exercise, and CTSB cross the BBB to act directly on the brain, particularly in the hippocampus [231,302]. Further research has shown that conditioned medium from skeletal muscle cells treated with the AMP-activated protein kinase (AMPK) agonist AICAR contains elevated levels of CTSB, and sustained exercise significantly increases CTSB concentrations in the gastrocnemius muscle and plasma of mice. Notably, CTSB knockout mice do not show exercise-induced improvements in cognitive function, suggesting that CTSB is a key mediator of the neuroprotective effects of exercise [231]. CTSB also confers neuronal protection through mechanisms such as reducing neuroinflammation [303,304] and promoting mitochondrial health [305]. Concurrently, it also enhances neurogenesis and cell proliferation by upregulating the expression of BDNF, thereby improving hippocampal structure and function [231].

BDNF is a pleiotropic neurotrophic factor that plays an important protective role in both the cardiovascular and nervous systems. Recent studies show that decreased circulating BDNF levels are significantly associated with adverse clinical outcomes in patients with HF [271]. Concurrently, low serum BDNF levels have also been confirmed as a biomarker of poor prognosis in cardiovascular disease [272]. BDNF, by activating its receptor tropomyosin receptor kinase B (TrkB), promotes neuronal growth, differentiation, survival, and synaptic regulation through mechanisms involving ERK, PI3K/AKT, and PLCγ pathways, while also modulating neuroinflammation and oxidative stress, inhibiting apoptosis, and supporting axonal myelination to sustain neuronal survival [306,307,308,309]. Peripheral BDNF concentration changes are considered partially derived from central nervous system release [273,274]. Exercise can promote BDNF expression through cholinergic drive in the hippocampus, or activate the hippocampal PGC-1α/FNDC5/BDNF pathway, alleviate mitochondrial oxidative stress, and improve cognitive impairment [232,310]. Significantly, exercise elevates plasma BDNF levels while attenuating post-infarction activation in the PVN and RVLM, thereby suppressing cardiac sympathetic overactivation [163].

#### 5.4.2. Hepatokine

Exercise also promotes the secretion of hepatocyte-derived cytokines that act on central neural circuits to improve cardiac function. Fibroblast growth factor 21 (FGF21) is primarily synthesized in the liver [311,312]. It crosses the BBB to act directly on the hypothalamus, regulating lipolysis, gluconeogenesis, fatty acid oxidation, and HPA axis activity [313]. FGF21 also directly activates noradrenergic neurons in the locus coeruleus (LC) [314]. Through the ventral tegmental area (VTA)-LC circuit, FGF21 modulates liver function, suppresses inflammatory responses and fibrosis, and promotes angiogenesis, thereby contributing to the repair of the infarcted heart [315]. These findings suggest that FGF21 serves as a key hepatokine linking exercise, brain function, and cardiac protection within the liver–brain–heart axis.

Glycosylphosphatidylinositol-specific phospholipase D1 (GPLD1) is an enzyme involved in the degradation of glycosylphosphatidylinositol and is produced mainly in the liver [316]. Exercise upregulates circulating GPLD1 levels, which has been correlated with improved cognitive function in mice [317]. Human studies further demonstrate that healthy, active elderly individuals have significantly higher blood GPLD1 concentrations than their sedentary counterparts [317]. Acute strength or aerobic exercise significantly increases serum GPLD1 levels, with no sex- or age-related differences in this response [275]. Mechanistically, GPLD1 may exert neuroprotective effects by promoting neuronal survival and regeneration, inhibiting apoptosis, and modulating the expression of inflammatory mediators and antioxidant enzymes [318,319]. In HF patients and mouse models, GPLD1 levels are significantly elevated; cardiac-specific GPLD1 knockout exacerbates cardiac dysfunction and myocardial hypertrophy, whereas overexpression ameliorates these adverse effects [320]. It is hypothesized that GPLD1 might indirectly influence brain function by modulating liver-derived factors (such as ketone bodies and other metabolites), which are transported to the brain via the bloodstream and subsequently support neuronal health and function [321]. Recent studies have confirmed that exercise-induced GPLD1 reverses aging- and Alzheimer’s disease-related memory loss by targeting the brain vasculature [322]. Overall, these studies highlight the liver as an active endocrine organ in exercise-mediated brain–heart crosstalk, with hepatokines such as FGF21 and GPLD1 playing central roles in linking exercise to neuroprotection, autonomic regulation, and cardiac recovery.

**Figure 4:**
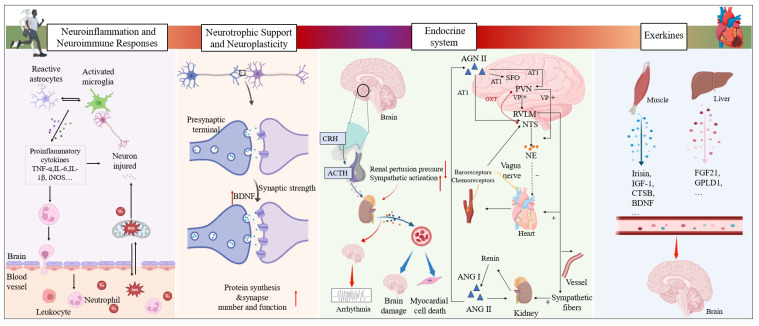
Potential central mechanisms by which exercise improves cardiovascular function via the brain–heart axis. Exercise improves cardiovascular function through several central pathways: by suppressing central inflammatory responses and regulating immune function, promoting neuronal plasticity, ameliorating neuroendocrine dysregulation and promoting the secretion of exerkines. ACTH adrenocorticotropic hormone, ANG I angiotensin I, ANG II angiotensin II, AT1 angiotensin II type 1 receptor, BDNF brain-derived neurotrophic factor, CRH corticotropin-releasing hormone, CTSB cathepsin B, FGF21 fibroblast growth factor 21, GPLD1 glycosylphosphatidylinositol specific phospholipase D1.

In summary, exercise confers cardioprotection in CVDs through coordinated central–peripheral regulation centered on the brain–heart axis. It suppresses neuroinflammation and oxidative stress within key autonomic nuclei, enhances neuroplasticity and neurotrophic support, and restores neuroendocrine and autonomic homeostasis. Concurrently, exercise-induced myokines and hepatokines function as critical humoral mediators that couple peripheral organ adaptation to central neural regulation. Through these integrated mechanisms, exercise re-establishes brain–heart communication, limits maladaptive neurohumoral activation, and alleviates cardiac dysfunction. Elucidating the molecular basis of this multidimensional crosstalk may open new avenues for mechanism-based, exercise-inspired therapeutic strategies in CVDs (Figure 4).

## 6. How Different Exercise Modalities Influence the Brain–Heart Axis

The brain–heart axis is not a single anatomical pathway, but rather a multilayered integrative system composed of the central autonomic network, the neuroendocrine system, immune-inflammatory signaling, the neurovascular unit, and the electromechanical activity of the heart [323,324,325]. Within this framework, the regulatory effects of exercise on the brain–heart axis cannot be simply described as being beneficial either to the brain or to the heart alone. Instead, exercise modifies the bidirectional coupling between the brain and the heart by influencing central plasticity, autonomic output, the peripheral metabolic environment, and cardiovascular adaptation.

### 6.1. Aerobic Exercise

Aerobic exercise is one of the most widely studied forms of physical activity and typically includes interventions such as running, swimming, and cycling. Owing to its favorable tolerability and broad systemic effects, this mode of exercise has been widely applied in models of neurological disorders and metabolic dysfunction. A randomized controlled trial by Hill et al. showed that a single bout of moderate-intensity aerobic exercise was sufficient to enhance neuroplasticity in the contralesional hemisphere of patients with stroke, suggesting that even acute aerobic stimulation can induce remodeling of central excitability [326]. Similarly, moderate- to high-intensity aerobic training has been shown to increase peripheral BDNF levels in patients with subacute stroke, indicating that regular aerobic exercise may promote neural recovery by enhancing neurotrophic support [327]. In older adults, exercise training lasting at least 4 weeks significantly increased resting BDNF levels, with moderate- to high-intensity protocols showing more stable effects [328]. In addition, the neuroprotective effects of aerobic exercise also involve stabilization of the cerebrovascular interface. In models of Alzheimer’s disease, cerebral small vessel disease, and chronic cerebral hypoperfusion, sustained aerobic exercise has been shown to protect the vascular systems of the hippocampus and cortex, delay BBB deterioration, and alleviate cognitive impairment [329,330]. More recent evidence suggests that the exercise-induced liver-derived exerkine GPLD1 can act on targets located on the cerebrovascular surface, thereby improving BBB integrity, reducing neuroinflammation, and reversing cognitive deficits associated with aging and Alzheimer’s disease [322]. Moderate-intensity aerobic exercise also improves autonomic balance and enhances the stability of cardiac rhythm. Regular exercise has been shown to improve HRV, including SDNN, RMSSD, and HF, suggesting enhanced parasympathetic activity and improved sympathovagal balance [331,332].

In contrast to moderate-intensity aerobic exercise, high-intensity aerobic exercise is more likely to amplify neurohumoral responses and cardiopulmonary stimulation within a shorter period, thereby inducing more pronounced adaptation of the brain–heart axis [333,334]. Compared with moderate-intensity protocols, high-intensity aerobic training more effectively improves peak oxygen uptake, enhances cardiac output reserve, and amplifies lactate signaling, catecholamine responses, and neurotrophic factor release. Therefore, in theory, it may be more effective in rapidly enhancing central plasticity and autonomic regulation [334,335]. However, these advantages generally depend on adequate exercise tolerance and recovery capacity. High-intensity aerobic exercise may amplify central responses through greater metabolic stress and stronger peripheral signaling. The protective effects of endurance exercise on healthy brain aging are closely associated with improvements in cerebral blood flow, reductions in neuroinflammation, upregulation of neurotrophic factors, and enhanced cardiorespiratory fitness, with cardiorespiratory fitness itself serving as an important mediator of brain protection. This suggests that when high-intensity aerobic training produces greater improvements in CRF, its potential neuroprotective effects may also be more evident [333]. Nevertheless, it should be emphasized that stronger physiological stimulation does not necessarily translate into better outcomes for the brain–heart axis, because excessive workload may also increase autonomic fluctuation, fatigue accumulation, and the risk of insufficient recovery.

### 6.2. Resistance Exercise

Resistance exercise exhibits biological characteristics in the regulation of the brain–heart axis that are clearly distinct from those of aerobic exercise. Its primary advantage does not lie in continuously improving cardiorespiratory fitness, but rather in increasing muscle mass, improving glucose and lipid metabolism, promoting the release of myokines and neurotrophic factors, and enhancing peripheral functional reserve, thereby providing important peripheral support for the brain–heart axis [336,337,338]. In other words, resistance exercise contributes to the regulation of the brain–heart axis more through a “muscle–metabolism–neurotrophic” pathway than through a predominantly “cardiopulmonary–autonomic” pathway [336].

Resistance training has been shown to improve the neurotrophic environment. It can promote the release of neuroprotective factors such as IGF-1, BDNF, and VEGF, while also alleviating depressive symptoms, and these effects may show a certain degree of dose dependence [336,337]. This suggests that resistance exercise does not act solely on skeletal muscle, but may also contribute to the maintenance of brain function through neurotrophic and cerebrovascular-related pathways. This is particularly relevant to the brain–heart axis, because cognitive decline, emotional disturbance, and autonomic imbalance often coexist, and the improvement in the neurotrophic milieu induced by resistance training may provide sustained neuroprotective support. In addition, the effects of resistance exercise on the brain–heart axis show clear acute–chronic differences. Acute resistance exercise, especially protocols involving large muscle groups, high loads, and short rest intervals, may induce sympathetic activation and transient increases in blood pressure. In contrast, long-term, regular resistance training may improve HRV, reduce resting sympathetic tone, and optimize overall autonomic flexibility [339,340,341]. In hypertensive populations, both aerobic and resistance training have been shown to improve indices related to sympathetic activity, although their modes of action are not entirely identical [341]. Therefore, resistance training may enhance the peripheral support capacity of the brain–heart axis by strengthening muscle–metabolic reserve and neurotrophic support. However, in individuals with hypertension, severe autonomic dysregulation, or unstable cardiovascular status, greater attention should be paid to load control and recovery scheduling.

### 6.3. High-Intensity Interval Exercise

High-intensity interval training (HIIT) has become one of the most intensively studied exercise modalities in recent years. This mode of exercise is characterized by repeated bouts of relatively high-intensity stimulation completed within a short period of time, thereby eliciting strong neurovascular stimulation with greater time efficiency [342]. Considerable evidence has accumulated regarding the effects of HIIT on neurotrophic factors and cognitive function. HIIT appears to be the most effective training modality for acutely increasing peripheral BDNF levels in adults, and this advantage may also be maintained during long-term interventions [342]. The BDNF released in response to HIIT may improve neurovascular coupling and promote more efficient regulation of cerebral blood flow in response to neural activity [343]. At the same time, HIIT exerts beneficial effects on cognitive domains such as executive function, memory, and information processing [344,345]. A single bout of HIIT has been shown to improve inhibitory control and working memory in healthy young adults, and may selectively modulate attentional network function; these changes appear to be partly associated with parasympathetic withdrawal and alterations in arousal level [346,347]. Therefore, HIIT may trigger central plasticity and cognition-related signaling through relatively large physiological perturbations.

In addition, HIIT often produces greater improvements in autonomic regulation and cardiorespiratory fitness than traditional training. HIIT ranks highly in improving HRV indices such as SDNN, RMSSD, and LF/HF [348]. In patients with heart failure or stroke, HIIT is often more effective than moderate-intensity continuous training (MICT) in improving VO_2_ peak and certain functional outcomes [349,350]. HIIT is comparable to, or even more effective than, moderate-intensity aerobic exercise in promoting angiogenesis [351] which may make it particularly suitable for patients in the recovery phase of stroke or ischemic disease. Compared with moderate-intensity aerobic exercise or combined training, the regulatory effects of HIIT appear to be more strongly oriented toward activating pathways related to angiogenesis and functional reconstruction, with greater emphasis on barrier repair rather than broad maintenance of systemic homeostasis [352]. However, because HIIT imposes a high physiological load, it may also pose challenges to inflammatory status and neural tolerance, thereby creating potential risks in some populations. Its short-duration but intense stimulation may lead to greater acute fluctuations in blood pressure, abrupt increases in heart rate, and higher recovery demands. Therefore, in individuals with poor baseline fitness, elevated arrhythmia risk, or limited recovery capacity, HIIT should be prescribed cautiously and accompanied by close monitoring.

### 6.4. Combined Exercise

Combined exercise generally refers to the integration of aerobic and resistance training, but it may also be extended to multicomponent interventions that incorporate balance, flexibility, or cognitive training. From the perspective of the brain–heart axis, the importance of combined exercise lies in its potential to simultaneously target multiple nodes, including central plasticity, autonomic output, muscle–metabolic support, vascular function, and long-term maintenance of physical capacity. Therefore, compared with any single exercise modality, it is more consistent with the biological nature of the brain–heart axis as a multi-link coupled system.

Available evidence suggests that combined exercise often yields favorable overall benefits at the cardiovascular and autonomic levels. Wang et al. [341] reported that, in patients with hypertension, the combination of aerobic and resistance training showed advantages in blood pressure regulation and in improving indices related to sympathetic activity. A systematic review by Zhang et al. [353] further showed that concurrent aerobic and resistance training can improve cognitive health, with more pronounced effects in older adults and clinical populations. Muñoz-Perete et al. [354] and Han et al. [355] further noted that the combination of physical training and cognitive stimulation improves cognitive performance in older adults with mild cognitive impairment, and that combined protocols with a greater exercise component tend to yield better outcomes. Although these studies do not always directly assess cardiac outcomes, the brain–heart axis fundamentally emphasizes the bidirectional coupling among central, circulatory, and peripheral functions. Therefore, in populations with cardiovascular risk factors, reduced physical capacity, or cognitive decline, combined exercise may be more suitable than a single modality for achieving concurrent improvements in cognition, autonomic regulation, and physical function.

### 6.5. The Paradox of Exercise: Potential Risks and the U-Shaped/J-Shaped Curve in the Brain–Heart Axis

Although regular exercise is unequivocally associated with broad cardiovascular benefit, its protective effects are unlikely to increase indefinitely with escalating exercise dose. Accumulating evidence suggests that, in a subset of individuals exposed to very high training volumes, prolonged endurance exercise, or repeated bouts of extreme-intensity exercise, the relationship between exercise and cardiovascular risk may follow a U-shaped or J-shaped curve [356,357,358]. In this setting, the so-called exercise paradox does not imply that exercise is intrinsically harmful; rather, it suggests that when exercise load exceeds an individual’s adaptive reserve, physiological remodeling may shift towards maladaptive neurocardiac stress responses.

Within the framework of the brain–heart axis, one of the most plausible mechanisms underlying this paradox is extreme sympathetic overactivation. Acute strenuous exercise is a potent physiological stressor that engages the sympathetic–adrenal system and the HPA axis to maintain cardiovascular and metabolic homeostasis [359]. Under appropriate conditions, this response is adaptive. However, when exercise is excessive in intensity, prolonged in duration, or insufficiently matched by recovery, sympathetic activation may become amplified and sustained, thereby disturbing central autonomic regulation rather than refining it [359]. The CeA, PVN, and NTS are involved in this process. Among them, the CeA is selectively activated during high-intensity exercise and promotes sympathetic drive. Lesions of the CeA prolong exercise duration and delay the sharp rise in blood pressure before fatigue, suggesting that this region may serve as a central “brake” on exercise performance [360]. Consistent with this concept, HRV-based analyses have shown that exhaustive exercise markedly reduces RMSSD, pNN50, and HF, while increasing LF/HF, indicating vagal withdrawal and relative sympathetic dominance [361]. From a brain–heart axis perspective, such findings support the view that excessive exercise may shift central autonomic control towards a state of persistent sympathoexcitation, thereby undermining neurocardiac homeostasis.

A second, closely related mechanism is stress hormone imbalance. Excessive exercise can provoke repeated surges in catecholamines and glucocorticoids, and chronic exposure to such neuroendocrine stress may no longer remain adaptive [362]. A recent systematic review and meta-analysis demonstrated that elevated norepinephrine, epinephrine, and cortisol levels are each associated with increased cardiovascular risk [362]. These observations are particularly relevant to the brain–heart axis, as sustained activation of stress hormone pathways may reinforce central stress circuitry, impair autonomic recovery, and heighten myocardial electrical and metabolic vulnerability. Thus, the adverse effects of excessive exercise may arise not merely from increased mechanical cardiac workload, but from the emergence of a maladaptive neuroendocrine milieu characterized by prolonged sympathetic predominance and dysregulated stress signaling.

This neuroautonomic burden is mirrored by acute cardiac perturbations observed after extreme endurance exercise. Studies of ultramarathon and ultra-endurance events have consistently reported transient elevations in cardiac biomarkers, including hs-cTnI and NT-proBNP, together with short-term declines in cardiac performance, particularly involving the right ventricle [363]. In a 130-km ultramarathon cohort, right ventricular fractional area change was significantly reduced after the race and remained depressed several days later in a subset of participants, especially in those with greater biomarker release [363]. Although such changes are often reversible, they nevertheless indicate that extreme exercise can transiently push the heart into a state of disproportionate stress. Mechanistically, this may reflect a convergence of hemodynamic overload, excessive sympathetic drive, and incomplete autonomic recovery, all of which are highly relevant to the destabilization of the brain–heart axis.

With chronic exposure, concern shifts from transient functional stress to structural and electrical remodeling. Moderate exercise is generally associated with a lower burden of atrial fibrillation, whereas long-term high-intensity endurance exercise may increase atrial fibrillation susceptibility through a combination of atrial enlargement, fibrosis, inflammation, and autonomic imbalance [364]. Similarly, myocardial fibrosis in endurance-experienced athletes was independently associated with incident ventricular arrhythmias, raising concern that repeated exposure to extreme training loads may, in susceptible individuals, promote an arrhythmogenic substrate [365]. Ultramarathon runners exhibit significant atrial electrical remodeling and abnormal P-wave indices, which may be associated with an increased risk of arrhythmias [366]. These findings are highly relevant to the brain–heart axis, because both atrial and ventricular arrhythmogenesis are strongly modulated by autonomic tone. Persistent sympathetic excess, particularly when coupled with impaired vagal recovery, may therefore serve not only as a functional trigger but also as a permissive factor for maladaptive structural remodeling.

Another important aspect of the exercise paradox is the observation that lifelong high-volume endurance exercise, particularly in older men, may be associated with a higher burden of coronary artery calcification (CAC) [357]. Although the prognostic significance of this phenomenon remains debated, and calcified plaques in athletes may differ in stability from those in sedentary populations, these data nevertheless challenge an overly simplistic assumption that greater exercise exposure always confers greater vascular benefit [357]. Likewise, exercise-related sudden cardiac arrest (SCA) and sudden cardiac death (SCD) remain rare overall, yet they are not negligible in susceptible individuals [367,368]. Population-based data indicate that exercise-related SCA is uncommon at the population level [367], but contemporary evidence also underscores that the causes of exercise-related SCA/SCD vary by age, with inherited or concealed heart disease predominating in younger athletes and coronary disease becoming more relevant in older master athletes [368]. In such settings, excessive sympathetic activation and stress hormone disequilibrium may act as critical triggers superimposed upon an underlying vulnerable substrate.

In summary, for most individuals, regular moderate-to-vigorous exercise remains profoundly beneficial. However, in those exposed to extreme exercise loads or harboring latent cardiovascular vulnerability, excessive exercise may perturb the brain–heart axis through sustained sympathetic overactivation, stress hormone imbalance, incomplete autonomic recovery, and downstream structural-electrical remodeling. Clinically, greater emphasis should be placed on precision exercise.

## 7. Concluding Remarks and Future Perspectives

Although a substantial body of epidemiological evidence and clinical research has established exercise as a cornerstone of both primary and secondary prevention in CVDs, the central challenge in this field has shifted from determining whether exercise is beneficial to addressing a more complex clinical question: how to formulate a precise exercise prescription for a specific patient at the right time, namely, precision exercise medicine. Although the brain–heart axis provides an important biological basis for this concept, major barriers remain in translating these mechanistic insights into clinical strategies.

First, the lack of reliable biomarkers remains a major barrier to clinical translation. At present, no specific biomarker is available to monitor brain–heart axis responsiveness in a real-time and noninvasive manner. Future studies should therefore focus on identifying and validating biomarkers that reflect neuroimmune interactions induced by exercise interventions. For example, could frequency-domain analysis of heart rate variability (HRV) serve as a dynamic indicator of exercise-induced vagal activation? Could specific microRNAs in plasma exosomes, such as miR-146a and miR-21, function as noninvasive liquid biopsy markers for quantifying the suppressive effects of exercise on neuroinflammation? Establishing quantitative relationships among exercise dose, biomarker dynamics, and cardiovascular outcomes will be a key scientific task for achieving individualized adjustment of exercise prescriptions.

Second, disease-stage specificity may determine the dual nature of exercise interventions. Our findings suggest that neural feedback mechanisms differ substantially across pathophysiological states. In patients with advanced heart failure, sympathetic activity is already markedly elevated, and excessive exercise loading may fail to restore autonomic balance and instead aggravate pathological cardiac remodeling through increased oxidative stress [369]. For such patients, very low-intensity exercise or breathing-based interventions aimed at neuromodulation may be more appropriate, with priority given to enhancing vagal tone. In contrast, during the early stages of hypertension or atherosclerosis, when sympathetic sensitivity remains modifiable, moderate- to high-intensity aerobic exercise may more effectively suppress sympathetic outflow and promote endothelial progenitor cell mobilization, thereby slowing disease progression [370]. Accordingly, future studies should employ randomized controlled designs across different stages of CVDs and explore differential exercise regimens stratified by baseline autonomic function, such as resting heart rate and baroreflex sensitivity.

In clinical practice, exercise interventions are rarely implemented in isolation, but rather alongside standard pharmacological therapies such as beta-blockers, angiotensin-converting enzyme inhibitors, and statins. At present, however, little is known about the molecular interactions between these medications and exercise, representing another major blind spot in precision exercise medicine. Future research should therefore incorporate exercise–drug interactions into the core framework of mechanistic investigation. On the one hand, certain medications may synergistically enhance the benefits of exercise. For example, although beta-blockers help control heart rate, they may also blunt the physiological heart rate response to exercise [371]. Meanwhile, newer agents such as sodium–glucose cotransporter 2 (SGLT2) inhibitors, which may improve myocardial energy metabolism and neural remodeling, could potentially exert additive or even synergistic effects when combined with exercise [372]. This raises the intriguing possibility that such agents may partially function as “exercise mimetics” in patients who are unable to tolerate high-intensity exercise. On the other hand, potential antagonistic interactions should not be overlooked. For instance, long-term statin use has been associated with mitochondrial dysfunction [373], which may attenuate exercise-induced mitochondrial biogenesis in skeletal muscle and myocardium. These mechanistic uncertainties suggest that medication use should be treated as a key stratification variable in both future clinical trial design and individualized exercise prescription.

In summary, our mechanistic findings highlight the central role of the brain–heart axis in mediating the cardiovascular benefits of exercise. However, to bridge the gap between mechanism and clinical application, a new paradigm of precision exercise medicine must be established. We propose that future research should focus on the following three directions. First, priority should be given to the development of a dynamic brain–heart axis monitoring system based on multimodal data integration. High-precision wearable devices could be used to continuously capture peripheral effector signals of the brain–heart axis, including heart rate variability, electrodermal activity, and accelerometer-derived micromovement features. Combined with circulating biomarkers such as neuroimmune-related miRNAs and inflammatory mediators, and supported by artificial intelligence algorithms, these multimodal data may enable noninvasive, continuous, and dynamic inference of the functional state of key nodes within the central autonomic network, particularly the insula, anterior cingulate cortex, and amygdala. On this basis, a digital exercise monitoring platform could be developed to assess brain–heart axis responsiveness and safety in real time, thereby providing a new technological pathway for precision exercise medicine. Second, stratified clinical trials are needed. Traditional homogeneous randomized controlled trial designs should be replaced or complemented by stratified approaches that distinguish among different CVDs stages, such as post-acute myocardial infarction, chronic stable heart failure, and early hypertension, as well as different autonomic phenotypes, such as high-sympathetic-tone and low-vagal-tone subtypes. Such studies are essential for determining the optimal exercise modality, intensity, and dose for each subgroup. Third, a drug–exercise interaction database should be established. Through large-scale real-world data analyses or combined drug–exercise intervention trials, future work should clarify the modifying effects of commonly used cardiovascular medications on exercise efficacy and construct an interaction map linking drugs, exercise, and outcomes. This would provide the foundation for integrated precision prescriptions that account simultaneously for pharmacological treatment and physical intervention. Only through such in-depth translational research can the broad concept that “exercise is medicine” be transformed into a cardiovascular treatment strategy that is as precise, quantifiable, and safe as pharmacological prescribing.

## Figures and Tables

**Table 1:** Exercise targets specific brain regions within the brain–heart axis: Neural effects and cardiovascular consequences.

Brain Regions	Cardiovascular-Related Potential Function	Potential Cardiovascular Benefits Mediated by This Region
PFC	Memory [144]; emotion [145]; cognition [146]	Amelioration of post-MI depression [112,136,137]; improved autonomic modulation [147]
IC	Depression; emotion [148]	Attenuation of arrhythmia risk [149]; prevention of cardiac injury [148,150,151,152]
Primary Motor Cortex (M1)	Movement execution, higher cognitive processes [153]	Cardiac control via MnR [6]; regulation of premature ventricular contractions post-MI [34]
Hippocampus	Memory [154], cognition [38]	Reduced atherosclerosis risk [155]; improved cognitive outcomes post-MI [156]
SFO	Fluid balance	Regulation of neurohumoral mechanisms [157,158]; inhibited sympathoexcitation during chronic heart failure [159]
Amygdala	Memory [160], depression	Slow down the progression of atherosclerosis [110]; mitigated stress-induced high blood pressure [161]
PVN	Stress	Decreased central sympathetic outflow [46,162,163], attenuated blood pressure in hypertension [164]; mitigated myocardial ischemia–reperfusion injury [165]
VMH	Emotion	Attenuated HR and BP [166,167]; reduced sympathetic outflow [168,169,170]
PAG	Stress response [171]	Myocardial infarction [172]; decreased sympathetic nerve activity and BP [173,174]
Nucleus of the solitary tract (NTS)	Pain [175]	Inhibition of stress-induced hypertension [176]
RVLM	A center for sympathetic regulation [177]	Attenuated BP in hypertension [164,178], decreased central sympathetic outflow [66]

BP Blood pressure, amygdala, HR Heart rate, IC Insular cortex, NTS nucleus of the solitary tract, PAG periaqueductal gray, PFC prefrontal cortex, PVN paraventricular nucleus, RVLM rostral ventrolateral medulla, SFO subfornical organ, VMH ventromedial hypothalamus.

## Data Availability

All data from this study are included in this article.

## Abbreviations

ACE, angiotensin-converting enzyme; Ach, acetylcholine; AChR, α7 nicotinic acetylcholine receptor. ACTH, adrenocorticotropic hormone; AMPA, α-amino-3-hydroxy-5-methyl-4isoxazolepropionic acid; AMPK, AMP-activated protein kinase; Ang II, angiotensin II; AT1R, angiotensin II type 1 receptors; AVP, arginine vasopressin; BBB, blood–brain barrier; AP, area postrema; BDNF, brain-derived neurotrophic factor; BNST, bed nucleus of the stria terminalis; CAIP, cholinergic anti-inflammatory pathway; CaMKII, Ca^2+^/calmodulin-dependent protein kinase II; CeA, the central amygdaloid nucleus; CREB cAMP response element binding protein, CRH, corticotropin-releasing hormone; CSAR, cardiac sympathetic afferent reflex; CTSB, Cathepsin B; CVDs, cardiovascular diseases; eNOS, endothelial NOS; EVs, extracellular vesicles; FFA, free fatty acids; FGF21, fibroblast growth factor 21; fMRI, functional magnetic resonance imaging; FNDC5, fibronectin type III domain-containing protein 5; fNIRS, functional near-infrared spectroscopy; GABA, Gamma-aminobutyric acid; Glu, Glutamate; GPLD1, Glycosylphosphatidylinositol-specific phospholipase D1; GR, glucocorticoid receptor; HbO, oxygenated hemoglobin; HF, heart failure; HIIT, high-intensity interval training; HPA, hypothalamic–pituitary–adrenal; HR, heart rate; HRV, heart rate variability; I/R, ischemia/reperfusion; ICH, intracerebral hemorrhage; IGF-1, insulin-like growth factor 1; IL-1β, interleukin-1β; IL-6, interleukin-6; iNOS, inducible NOS; LepR, leptin receptors; LC, locus coeruleus; LPBN, the lateral parabrachial nucleus; LPS, lipopolysaccharide; LSG, left stellate ganglion; LVEF, left ventricular ejection fraction; M1, the primary motor cortex; MAP, mean arterial pressure; MI, myocardial infarction; MIRI, myocardial ischemia/reperfusion injury; MnR, the median raphe nucleus; PFC, the medial prefrontal cortex; MS, multiple sclerosis; NADPH, nicotinamide adenine dinucleotide phosphate; NE, norepinephrine; NF-Κb, nuclear factor kappa-B; NMDA, N-methyl-D-aspartic acid; nNOS, neuronal NOS; NO, nitric oxide; NOS, nitric oxide synthase; Nrf2, nuclear factor erythroid 2-related factor 2; NTS, nucleus tractus solitarius; PBN, The parabrachial nucleus; PFC, the prefrontal cortex; PICs, proinflammatory cytokines; PGC-1α, proliferator-activated receptor-γ coactivator-1α; PLC-γ phospholipase C-gamma, PPAR-γ, peroxisome proliferator-activated receptor-γ; PVN, hypothalamic paraventricular nucleus; RAS, renin–angiotensin system; ROS, reactive oxygen species; RSNA, renal sympathetic nerve activity; RVLM, the rostral ventrolateral medulla; SAH, subarachnoid hemorrhage; SCFAs, short-chain fatty acids; SFO, the subfornical organ; SHR, spontaneously hypertensive; SOD1, superoxide dismutase; SpNS, stimulation of the splenic nerve; TBI, traumatic brain injury; tDCS, transcranial direct current stimulation; TLR4, Toll-like receptor 4; TMAO, trimethylamine N-oxide; TNF-α, tumor necrosis factor-α; TrkB, tropomyosin-related receptor kinase Bα7n.
